# Machine learning of atomic dynamics and statistical surface identities in gold nanoparticles

**DOI:** 10.1038/s42004-023-00936-z

**Published:** 2023-07-05

**Authors:** Daniele Rapetti, Massimo Delle Piane, Matteo Cioni, Daniela Polino, Riccardo Ferrando, Giovanni M. Pavan

**Affiliations:** 1 Department of Applied Science and Technology, Politecnico di Torino, Corso Duca degli Abruzzi 24, 10129 Torino, Italy; 2 Department of Innovative Technologies, University of Applied Sciences and Arts of Southern Switzerland, Polo Universitario Lugano, Campus Est, Via la Santa 1, 6962 Lugano-Viganello, Switzerland; 3 Department of Physics, Università degli Studi di Genova, Via Dodecaneso 33, 16146 Genova, Italy

**Keywords:** Condensed-matter physics, Theory and computation, Chemical physics, Statistical mechanics

## Abstract

It is known that metal nanoparticles (NPs) may be dynamic and atoms may move within them even at fairly low temperatures. Characterizing such complex dynamics is key for understanding NPs’ properties in realistic regimes, but detailed information on, e.g., the stability, survival, and interconversion rates of the atomic environments (AEs) populating them are non-trivial to attain. In this study, we decode the intricate atomic dynamics of metal NPs by using a machine learning approach analyzing high-dimensional data obtained from molecular dynamics simulations. Using different-shape gold NPs as a representative example, an AEs’ dictionary allows us to label step-by-step the individual atoms in the NPs, identifying the native and non-native AEs and populating them along the MD simulations at various temperatures. By tracking the emergence, annihilation, lifetime, and dynamic interconversion of the AEs, our approach permits estimating a “statistical equivalent identity” for metal NPs, providing a comprehensive picture of the intrinsic atomic dynamics that shape their properties.

## Abstract (web summary)

Metal nanoparticles are effective catalysts with properties very different from their bulk species, but insight into atom dynamics within nanoparticles is non-trivial to attain. Here, a data-driven approach elucidates the physical mechanisms underlying the temperature-dependent properties of gold nanoparticles.

## Introduction

Metal nanoparticles (NPs) exhibit properties significantly differing from their bulk counterparts due to their size, shape, surface, and dynamical features^1–4^. However, this requires obtaining detailed insight into their atomic structure and dynamics which are typically not easy to attain.

Gold (Au) NPs are a relevant example. Being the most stable among transition metals, bulk Au is often considered an inert catalyst. On the other hand, Au nanoparticles (Au NPs) are in comparison surprisingly active and effective catalysts^3–6^, capable, e.g., of oxidizing CO into CO_2_ in atmospheric conditions^4,7^, and of catalyzing various other oxidative transformations^4^. Notably, Au NPs also feature surface plasmon resonance (SPR)^2^ and several other distinct physical and chemical attributes, somehow directly related to the shape and features of their surface, which make them interesting candidates for sensor devices and biomedical applications^2,8^.

Metals, in general, are known to assume a non-trivial dynamic behavior, where atoms enter a dynamic steady state and can move in the lattice, well below their melting temperature^9–13^. Such atomic mobility allows dynamic transformations of the material’s surface and reconstruction of specific atomic environments^12,14–23^. This is even more relevant in the case of metal NPs, due to a well-known (albeit not general) dependence of their melting temperature with size^24,25^, meaning that smaller NPs may exhibit significant surface atomic mobility even at relatively low temperature^26^. Understanding the dynamic properties of the atomic sites that populate the surface of Au NPs and how these evolve and change in time in relevant conditions is thus of utmost importance. For example, it may facilitate the rational design of more effective NP-based heterogeneous catalysis strategies^27,28^. Indeed, seminal work in the field has underscored the importance of understanding the dynamic formation of transient active sites on the surface of Au NPs^29^. These sites, which form only under specific reaction conditions, play a crucial role in catalytic processes, underscoring the need for methods capable of accurately predicting and studying these transient sites and their dynamics. For this purpose, experimental investigation techniques have been recently developed, increasing the resolution up to single particles^30^ or even individual atoms^5,26^. For example, high-angle annular dark-field scanning transmission electron microscopy (HAADF-STEM) experiments of supported Au NPs provided direct evidence that atoms move in the NPs at finite temperatures^5,26^. However, unraveling such atomic motion and obtaining quantitative insight about it remains non-trivial for a series of fundamental reasons, including, e.g., the structural dispersion and variable distribution of atomic surface sites, which details get considerably smoothed out in ensemble-averaged characterizations^30^, or the fact that experimentally-reconstructed NP models do not contain information concerning the identity of the individual atoms (so that following their dynamics over time is impractical)^5,26^.

Widely used for studying metals and metal clusters^23,31–39^, computational modeling holds considerable potential in this sense. Molecular simulations have been used to study Au NPs with considerable success^5,40–44^. The intrinsic ability of atomistic simulations to capture individual atomic motions and track them over time is particularly advantageous for the reconstruction of the internal atomic dynamics of NPs^23,27^. Machine learning approaches were recently found very useful to analyze molecular dynamics (MD) trajectories of various types of complex molecular systems^23,45–49^, including Au NPs^41^. In particular, unsupervised clustering and advanced statistical analyses of high-dimensional smooth overlap of atomic positions (SOAP)^50,51^ data extracted from MD simulations recently allowed to reconstruct the structural/dynamical complexity of a variety of molecular materials/systems^23,45,46^ and to build robust data-driven metrics^48,49^ useful for their classification^47^.

In this work, we focus on the temperature-dependent properties of Au NPs and provide insights into the underlying physical mechanisms that drive their behavior in different conditions. To achieve this, we designed and employed an unbiased analysis pipeline that combines a bottom-up data-driven approach with a top–down dictionary-based approach that is described herein. This pipeline allows us to resolve at atomistic-resolution the complex dynamics and identify the statistical identities of metal NPs of different shapes and sizes in relevant conditions. We simulate various types of Au NPs (i.e., icosahedral, decahedral, octahedral) on relevant spatiotemporal scales. Our data-driven approach, based on SOAP features and clustering techniques, identifies and characterizes metastable states with greater accuracy and efficiency than previous methods. Particularly, a combination of bottom-up and top–down SOAP-based data-driven analyses reveal the atomic environments (AEs) that statistically populate Au NPs along the MD simulations at various temperatures, permitting tracking the native and non-native AEs—that is, AEs typical of other types of NPs—that continuously emerge/resorb on the NPs. This allows us to obtain precious information on their emergence, annihilation, survival lifetime, and dynamic interconversion, and a unique insight into how such innate atomic dynamics shape the “statistical identity" of the NPs in given conditions. By quantifying these properties, our analysis not only elucidates the fundamental behavior of NPs but can also serve as a predictive tool for their performance in various applications, e.g., catalysis, ultimately leading to more sustainable and cost-effective industrial processes.

## Results

### Characterizing the innate dynamics of a gold NP *via* machine learning of atomic environments

**Fig. 1:**
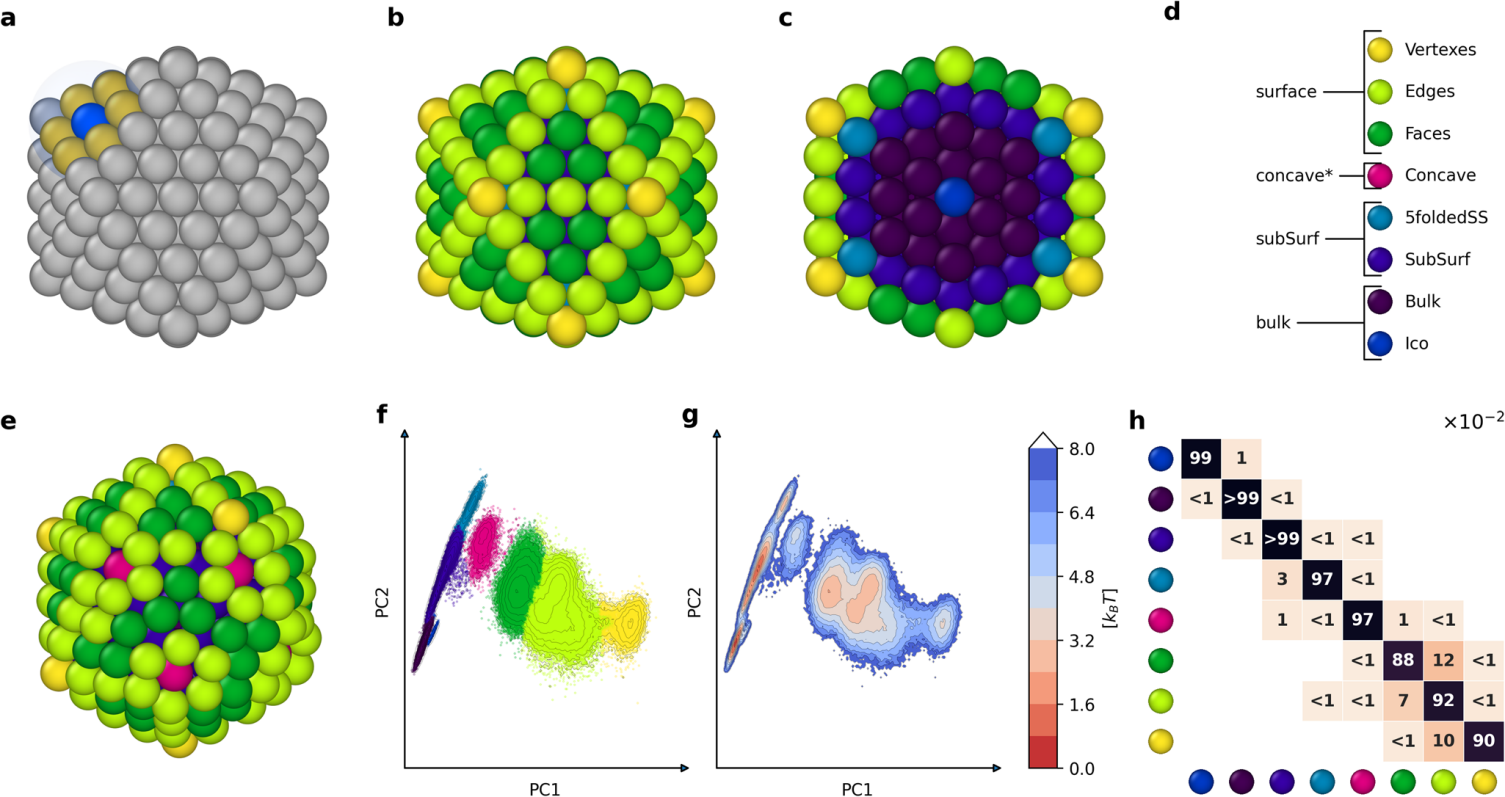
Bottom-up machine learning of atomic environments (AEs) and AEs’ dynamics in *Ih*_309_ at 300 K. **a** A SOAP vector is centered in each atom of the Au NP (in blue), obtaining a SOAP spectrum which is a characteristic fingerprint of the level of order/disorder in the displacement of the neighbor Au atoms (in gold) within a cutoff (shown as a transparent sphere). The SOAP spectra of all atoms in the NP (309) are calculated on 1000 frames taken every 1 ns along the last 1 μs of MD (see also Supplementary Fig. S6), obtaining a SOAP dataset containing 309,000 SOAP spectra in total. The main AEs that populate the *Ih*_309_ NP are identified *via* unsupervised clustering using the HDBSCAN* algorithm (see Methods section for details). **b**, **c** Main AEs present on the surface (**b**) and in the interior (**c**) of the ideal *Ih*_309_ NP before simulation starts (at 0 K). **d** Color legend showing a structural interpretation of the AEs detected by the SOAP clusters. **e** Snapshot of *Ih*_309_ taken from the MD simulation (at 2  μs) at 300 K, with Au atoms colored based on the detected SOAP clusters. **f** PCA projection on the two first principal components of the SOAP dataset. The different colors identify the various main clusters detected by HDBSCAN*. **g** The inverse logarithm of the density of the SOAP dataset, identifying the SOAP clusters (AEs) as local energy minima. **h** Normalized transition matrix reporting the probabilities for an atom in a given AE at time *t* to remain in that AE (*p*_*ii*_) or to undergo a transition to a different AE (*p*_*i*→*j*_) in *dt* (i.e., at *t* + *dt*, with *dt* = 1 ns in our analyses). All *p*_*ii*_ and *p*_*i*→*j*_ values in the matrix are × 10^−2^.

As a first representative example of ideal Au NPs, we investigate, analyze and reconstruct the innate atomic dynamics of a 309 atoms icosahedron (*Ih*_309_) at various temperatures. An icosahedral NP can be imagined as a series of concentric shells that envelop a single central atom. The first shell that resembles an icosahedron is constituted by the first 12 atoms surrounding the central one. Larger icosahedra can be generated by adding further surrounding atomic shells, while at each new larger shell, the NP resembles more and more the ideal platonic solid with 20 equilateral triangles as faces and 12 vertexes. Ideal atomic icosahedral NPs can be thus obtained as composed of 13, 55, 147, 309, 561, 923, etc., atoms)—the so-called “magic atomic numbers" for icosahedral NPs. As a relevant example, here we start by studying the behavior of an ideal Au icosahedral NP composed of 309 atoms (Fig. 1: *Ih*_309_) at different temperatures *via* classical molecular dynamics (MD) simulations.

As a first step, we built an atomistic model of *Ih*_309_ (Fig. 1a–c) that we simulated via 2 μs of MD at 300 K (see Methods section for complete computational details). From the resulting MD trajectories, we extracted 1000 frames sampled every 1 ns from the last 1 μs of MD (during which the population of the detected AEs plateau—MD steady state—see Supplementary Fig. S6). Recently proven useful to reconstruct the structural and dynamical complexity of various types of self-organizing molecular/atomic systems^23,45–47,49^, we used the smooth overlap of atomic positions (SOAP) vectors^50,51^ as abstract high-dimensional descriptors capable of retaining rich information on order/disorder in the AEs–i.e., in the local atoms’ displacement (Fig. 1a: in yellow) around each atom in the NP (in blue) within a cutoff. In particular, our SOAP analysis is performed using a cutoff of *rcut* = 4.48 Å, which was found as the best compromise between the cost of the calculation and the highest retained information in this case. We used SOAP rather than other geometrical analysis tools (such as polyhedral template matching^52^ or common neighbors analysis^53^), because it is a non discreet descriptor that can capture the local atomic environment more accurately and robustly than conventional methods that tend to ignore the surface details of the system. SOAP also has the advantage of being easier to extract data from it out of the box. These features make SOAP suitable for studying dynamic materials with diverse structures and properties. At each sampled frame (1000) during the MD simulation, we calculated the SOAP^50,51^ power spectrum for each atom (309) at that frame in the *Ih*_309_ NP, obtaining a global SOAP dataset composed of 309,000 SOAP spectra in total. From this SOAP dataset, we then identified the main AEs that populate the *Ih*_309_ NP at 300 K via unsupervised clustering using the HDBSCAN* algorithm (see Methods section for complete details)^54^.

This analysis finds eight different clusters (AEs) emerging in the *Ih*_309_ at 300 K (Fig. 1b–f). Particularly evident in Fig. 1b, c, the identified SOAP clusters correspond to different structural AEs on the *Ih*_309_ NP. In detail (Fig. 1d), we obtain an “*Ico*" AE, corresponding to the central atom of the icosahedral *Ih*_309_ NP (in blue). Shown in Fig. 1c, such AE is different from the “*Bulk*" AEs (in violet) surrounding it in the bulk of the NP (this 13 atoms AE is non-crystalline, i.e., it is not possible to cover the whole space by units of this AE). The analysis detects “*SubSurf*" and “*5foldedSS*" AEs, identifying the atoms in the first layer below the NP surface (deep blue) and vertexes (characterized by a 5-folded symmetry axis: in light blue). On the surface of the (ideal) *Ih*_309_ NP (Fig. 1b), our analysis detects a “*Faces*" (dark green)—close compact FCC(111) facet environments –, “*Edges*" (light green), and “*Vertexes*" AEs (the lowest coordinated atoms in the NP, in yellow). Shown in Fig. 1d, the analysis also detects an additional pink cluster, namely, a “*Concave*" AE identifying the centers of the so-called rosettes (Fig. 1e)^44^. Noteworthy, such concave AEs are not present in the ideal *Ih*_309_ NP (Fig. 1b) but they emerge along the MD (see also Supplementary Movie 1), while their formation is known to be an energetically favored event on the surface of icosahedral NPs^44,55^. Figure 1e shows a representative MD snapshot of the *Ih*_309_ at 300 K, where a rosette triplet—formation of rosettes pertaining to three neighbor vertexes, known experimentally—is clearly visible. Notably, each rosette center (pink) has six atom neighbors while classic vertexes (yellow) have five in a *Ih*_309_ NP. In particular, once a rosette triplet is formed, this configuration is found stable during the MD simulation, while even at 300 K, this is accompanied by continuous collective atoms motions that do not change the overall shape of the surface of the icosahedral NP (see Supplementary Movie 1).

Figure 1f shows the PCA (projection on the two first components PC1 and PC2) of the SOAP power spectra dataset, colored based on the SOAP clusters detected via unsupervised HDBSCAN* clustering. From the inverse logarithm of the PCA density, it is also possible to obtain the corresponding free energy surface of Fig. 1g. From these two plots, we can clearly distinguish three different zones on the PCA and obtain the first qualitative information on their interconnection. The clump of AEs on the left collects the bulk and subsurface environments (dark blue, violet, and light blue). These SOAP environments correspond to quite dense areas in the PCA, which indicates substantially low mobility at 300 K of the atoms that belong to these AEs in the *Ih*_309_ NP. The right part of the PCA is much less compact, indicating that the surface AEs (dark-, light green, and yellow AEs: faces, edges, and vertexes) are in comparison much more dynamic at 300 K. Between the bulk and surface areas, there is a smaller zone in the PCA connecting them. The vast majority of these environments are classified as “*Concave*" (pink), which suggests that at room temperature the interior and exterior of these NPs communicate essentially *via* the creation of local “point defects” created on their surface—if we think of rosettes in this sense, as they are not present in the ideal icosahedral NP. The free energy surface of Fig. 1g is derived from the density of points in each cluster, but it is not weighted by the population of each cluster. This means it does not fully represent the probability of an individual atom to visit the landscape. Nonetheless, this shows (i) how the various detected SOAP clusters (AEs) correspond to local density maxima and energy minima and (ii) that the barriers separating the surface states are relatively low, which allows for the considerable atomic exchange between these AEs.

Quantitative information on the internal dynamics of the NP can be obtained by tracking the SOAP spectra of all atoms at each sampled MD snapshot and monitoring their change. In particular, this allows us to analyze what SOAP AE each atom belongs to at time *t* and at each successive timestep (i.e., at *t* + *dt*, with *dt* = 1 ns in our analyses). Figure 1h shows a normalized transition matrix for the *Ih*_309_ NP at 300 K. This contains all probabilities (all values reported in the matrix are to be intended as multiplied by × 10^−2^) for an atom in a given AE *i* to remain in that AE (*p*_*ii*_) or to undergo a transition to a different AE (*p*_*i*→*j*_) in *dt* (the rows of the matrix sum to 1). Note that while such normalized transition matrices are non-symmetric (due to normalization), the corresponding raw matrices counting all transitions between the AEs observed along the MD are substantially symmetric (Supplementary Fig. S2), as it pertains to a microscopic equilibrium along the sampled MD regime (Supplementary Fig. S6). Figure 1h shows that at 300 K the deep/core AEs tend to be rather stable (*p*_*ii*_ ~ 1: atoms belonging to such AEs have a high probability of remaining in such state in *dt* = 1 ns). On the other hand, a significant inter-AE exchange is already observable (*p*_*i*→*j*_ ≥ 1) at this temperature on the NP surface. Indeed, the matrix shows that in such conditions most of the action takes place in the sub-square in the matrix connecting the ’*Faces*’, ’*Edges*’, and ’*Vertexes*’ AEs (dark-, light green, and yellow AEs).

From the transition probabilities of Fig. 1h, it is possible to estimate the average lifetime of the various AEs and the transition rates between them. In particular, the off-diagonal entries (*p*_*i*→*j*_) divided by *dt* give the transition rates between two AEs *i* → *j*, *k*_*i*→*j*_, from which one can estimate the characteristic timescales for the various transitions as: ${\tau }_{i\to j}={k}_{i\to j}^{-1}$. The number of times a given transition event *i* → *j* is registered in the system along the last 1000 ns is reported in Supplementary Fig. S2 as explicitly counted along the MD simulation, or it can be also estimated as: *n*_*i*→*j*_ = [*i*] ⋅ 1000/*τ*_*i*→*j*_, where [*i*] is the average number of atoms in the *i*^*th*^ AE (Supplementary Fig. S1). For example, in the *Ih*_309_ NP at 300 K an atom in the *Faces* AE (dark-green) has a transition probability to the *Edges* AE (light green) of *p*_*Faces*→*Edges*_ ~ 0.12 (~12 × 10^−2^), indicating a transition rate of *k*_*Faces*→*Edges*_ ~ 0.12 ns^−1^ and characteristic transition timescale *τ*_*Faces*→*Edges*_ ~ 8.3 ns (Supplementary Fig. S2 and S6: transition event observed ~6700 times along the last 1 μs of MD simulation). Furthermore, since this is the fastest transition involving the *Faces* AE, this sets the bottom limit for the lifetime of an atom in the (111) faces of this NP at 300 K (minimum residence time) as *τ*_*Faces*_ ~ 8.3 ns.

Similar estimations for other dynamic transitions between the AEs within the NP can be calculated from the transition matrix of Fig. 1h in an analogous way. We note that given the time window used for the analysis we report herein (*dt* = 1 ns), any observed communication/exchange between the AEs involves processes happening on the ns scale or slower, thus reducing the probabilities that the AE exchanges are related to thermal vibrations (values related to *p*_*i*→*j*_ < 0.01 (i.e., 1 × 10^−2^ in Fig. 1h) should be considered as purely qualitative, as these pertain to events that are only sparsely observed along the MD simulation). We also underline that, while the exact estimated values for AEs’ lifetimes, probabilities, and transition rates may slightly change depending on the employed FF^43,56–58^, tests conducted with different types of FFs^59,60^ provided very similar results in terms of NP dynamics, confirming the generality of our observations.

**Fig. 2:**
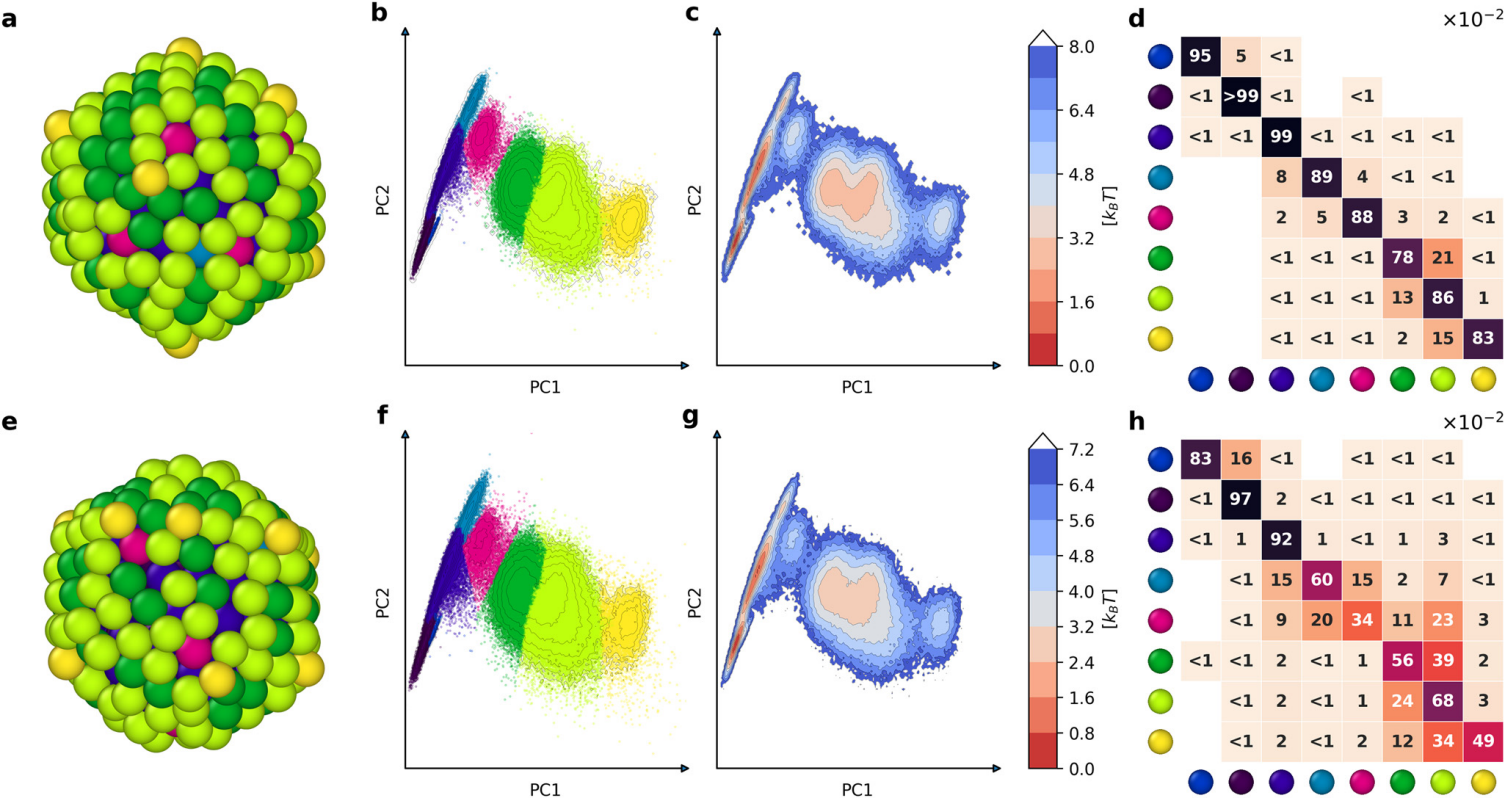
Effect of temperature on the *Ih*_309_ NP dynamics. **a** MD frame of the *Ih*_309_ NP taken from the equilibrated-phase MD simulation at 400 K (atoms colored based on SOAP clusters of Figure 1). **b** PCA projection of the SOAP dataset obtained from the MD simulation of the *Ih*_309_ NP at 400 K. **c** Free energy surface (FES) obtained from SOAP PCA. **d** Normalized transition matrix indicating the residence (*p*_*ii*_) and transition probabilities between the AEs in the *Ih*_309_ NP at 400 K of temperature in the time interval *dt* = 1 ns (all *p*_*ii*_ and *p*_*i*→*j*_ values are × 10^−2^). **e** MD frame of the *Ih*_309_ NP taken from the equilibrated-phase MD simulation at 500 K. **f** PCA projection of the SOAP dataset obtained from the MD simulation of the *Ih*_309_ NP at 500 K. **g** Associated free energy surface (FES). **h** Normalized transition matrix indicating the residence (*p*_*ii*_) and transition *p*_*i*→*j*_) probabilities ( × 10^−2^) between the AEs in the *Ih*_309_ NP at 500 K in the time interval *dt* = 1 ns.

We simulated the *Ih*_309_ NP also at 400 K and 500 K by running 2 μs of MD(see also Supplementary Movie 2). We then extracted the SOAP spectra for all atoms from 1000 frames taken from the last 1 μs of MD following the same protocol used at 300 K. Fig. 2 shows the results of these additional analyses. In particular, in these analyses, we used the simulation at 300 K as the training set for both the PCA computation and clustering (HDBSCAN*) analyses of the MD trajectories of the *Ih*_309_ NP at 400 K and 500 K (see Methods section for complete computational details). The PCAs of Fig. 2b, f show how the clusters on the surface of the NP become more adjacent to each other at 400 K and 500 K than at 300 K. Moreover, the FESs of Fig. 2c, g indicate that the minima corresponding to different surface AEs at 300 K tend to merge together when the temperature increases. In particular, at 500 K, the surface AEs constitute a unique large minimum, meaning that at such temperature, e.g., *Faces* and *Edges* AEs are in continuous exchange with each other and that these effectively form a unique fuzzy surface state (i.e., computing the PCA on the MD trajectory at 500 K would not find at all two distinct *Faces* and *Edges* AEs, but one single environment). The communication between *Faces*, *Edges*, and *Vertexes* AEs increases with increasing temperature. This is even more evident in the normalized transition matrices of Fig. 2d, h. The sub-square in the matrices connecting the ’*Edges*’, ’*Faces*’, and ’*Vertexes*’ AEs shows that atoms belonging to these environments have larger probabilities to exchange with each other at 400 K and 500 K than at 300 K. In particular, at 400 K, such a surface atomic mobility is evident, but these atoms have still a higher probability of remaining in their environment than of jumping into another one in *dt* = 1 ns (*p*_*ii*_ > 50%). On the other hand, at 500 K the residence probability for atoms in the surface AEs drops close to, and in some cases also below 50%, suggesting that in such conditions the NP surface is pre-melting^61^. We note that, in good approximation, the number of atoms in each environment does not vary much during the simulations at all temperatures (Supplementary Fig. S6). This suggests that, despite such rich atomic mobility, the *Ih*_309_ NP surface remains structurally that of an icosahedron at all analyzed temperatures. It is also interesting to note that the transition matrix of Fig. 2h shows sparsely observed dynamic interconnections between the central atom of the NP and the surface AEs at 500 K. This does not mean that the central atom is diffusing to the NP surface, but rather that at such temperature internal voids may rarely form in the NP center, which makes the SOAP spectrum of deep bulk atoms change occasionally and become similar to that of surface AEs. This fits well with previous reports showing similar central vacancies in *Ih*_309_^55,62^.

**Fig. 3:**
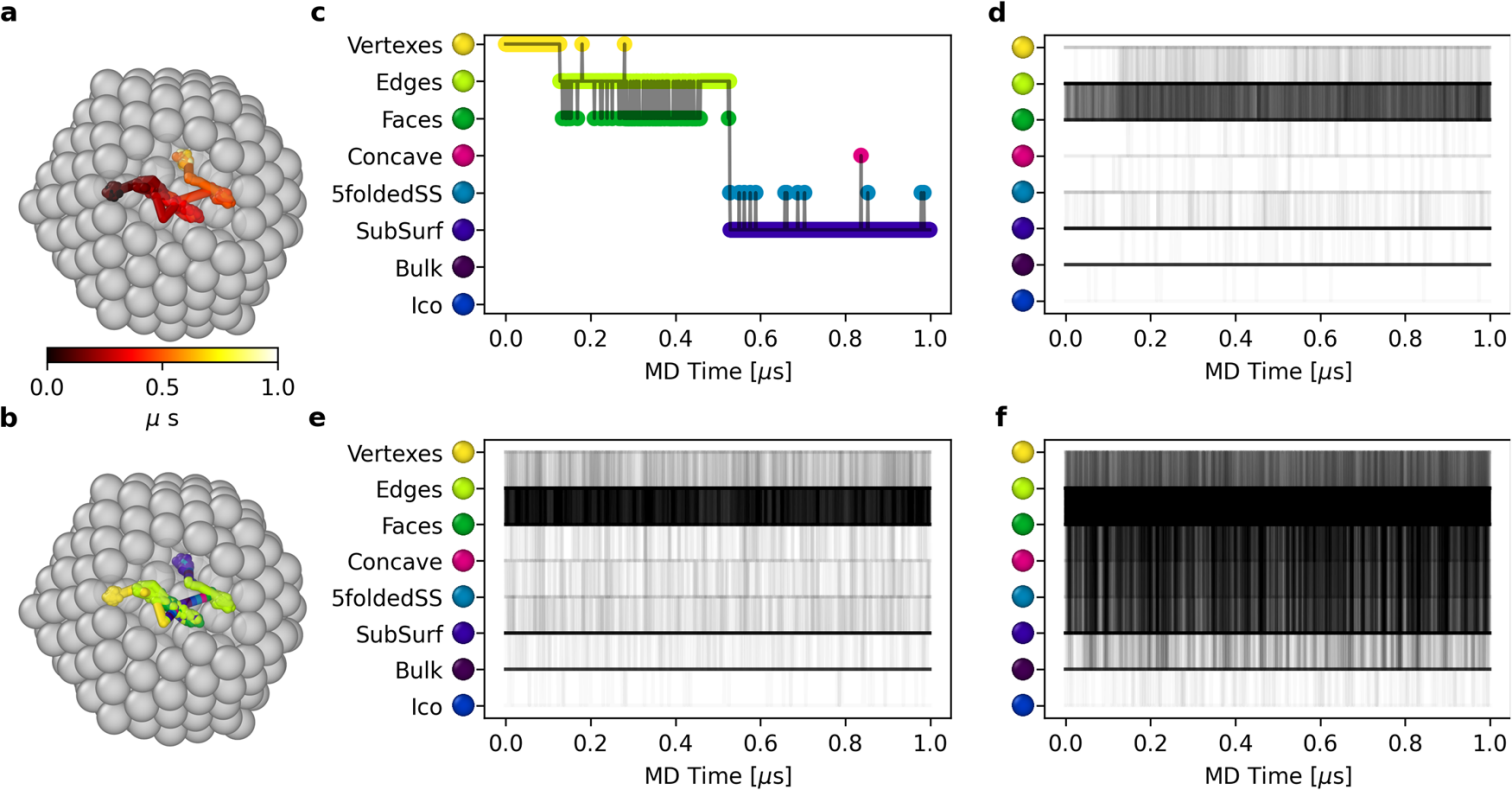
Atomic diffusion on the *Ih*_309_ NP. **a** MD trajectory of an atom in the *Ih*_309_ NP at 300 K, colored based on simulation time. **b** MD trajectory of the same atom colored based on its SOAP AE (Fig. 1): being initially a vertex, the atom diffuses on the NP surface visiting various surface and even subsurface AEs. **c** AEs visited by the tracked atom during the MD of the *Ih*_309_ NP at 300 K. **d** AEs' transitions of all (309) atoms in the *Ih*_309_ NP at 300 K: at room temperature, only the surface of the NP appears as dynamic. **e** AEs' atomic transitions in the *Ih*_309_ NP at 400 K. **f** AEs' atomic transitions in the *Ih*_309_ NP at 500 K: surface pre-melting.

While evidence of surface dynamics in Au NPs have been reported^5,27^, obtaining clear insights on the processes that characterize such dynamics, or on whether this is essentially due to, e.g., local atomic reconfigurations or atomic diffusion is non-trivial. Tracking the motions and the fluctuations in the SOAP spectra of the individual atoms in the NP, our approach provide clear evidence that the dynamics of these NPs is not due only to oscillations between adjacent/similar AEs, but to real microscopic atomic diffusion. As a representative example, in Fig. 3 we show the detail of the evolution during 1 μs of MD of an atom that is an *Ih*_309_ vertex at the start of the simulation. Figure 3a, b show respectively the temporal trajectory of the atom and the SOAP AEs that this visits in this time frame, revealing how even at 300 K such atom visits a surprisingly large collection of different (surface and subsurface) SOAP AEs (Fig. 3b). In particular, such vertex atom diffuses first to surface AEs, and then also penetrates into the subsurface. Its diffusion is described in detail also in the plot of Fig. 3c, showing how such an atom also becomes at a certain point (~820 ns of MD) a rosette center (pink).

Figure 3d–f show the AEs visited by all the 309 atoms in the NP at 300 K (d), 400 K (e), and 500 K (f). These graphs reveal which environments are most prone to exchange in this NP. In particular, at 300 K only *Faces* and *Edges* surface AEs are dynamic. At 400 K the dynamics of the NP surface increases, but remains similar to that at 300 K (which fits well with the transition matrices of Fig. 1h and Fig. 2d). On the other hand, at 500 K the atoms exchange between all surface and subsurface AEs (surface pre-melting).

We underline how all the analyses reported above are purely bottom-up, meaning that all information on the AEs, their similarity, classification, and dynamics are reconstructed only from the MD trajectories and in an unbiased data-driven way. At the same time, such data-driven analyses are not always straightforward to interpret. For example, given the surface of such NPs is in continuous motion and new non-native states may also emerge in these NPs (e.g., concave ones), a relevant question is whether such new non-native emerging AEs are closer to the native ones (proper of that type of ideal NP) or, e.g., to other AEs native of different types of NPs. To answer such questions and obtain a more complete picture, we employed a different type of analysis.

### A dictionary of Au NPs SOAP environments

To complement our study, we designed a different top–down analysis. We defined a “general" and transferable dictionary of SOAP environments analyzing ideal Au NPs (at 0 K) of different sizes and morphologies. We then used it to identify the native and non-native AEs that emerge in the simulated NPs and to analyze their dynamics at different temperatures.

**Fig. 4:**
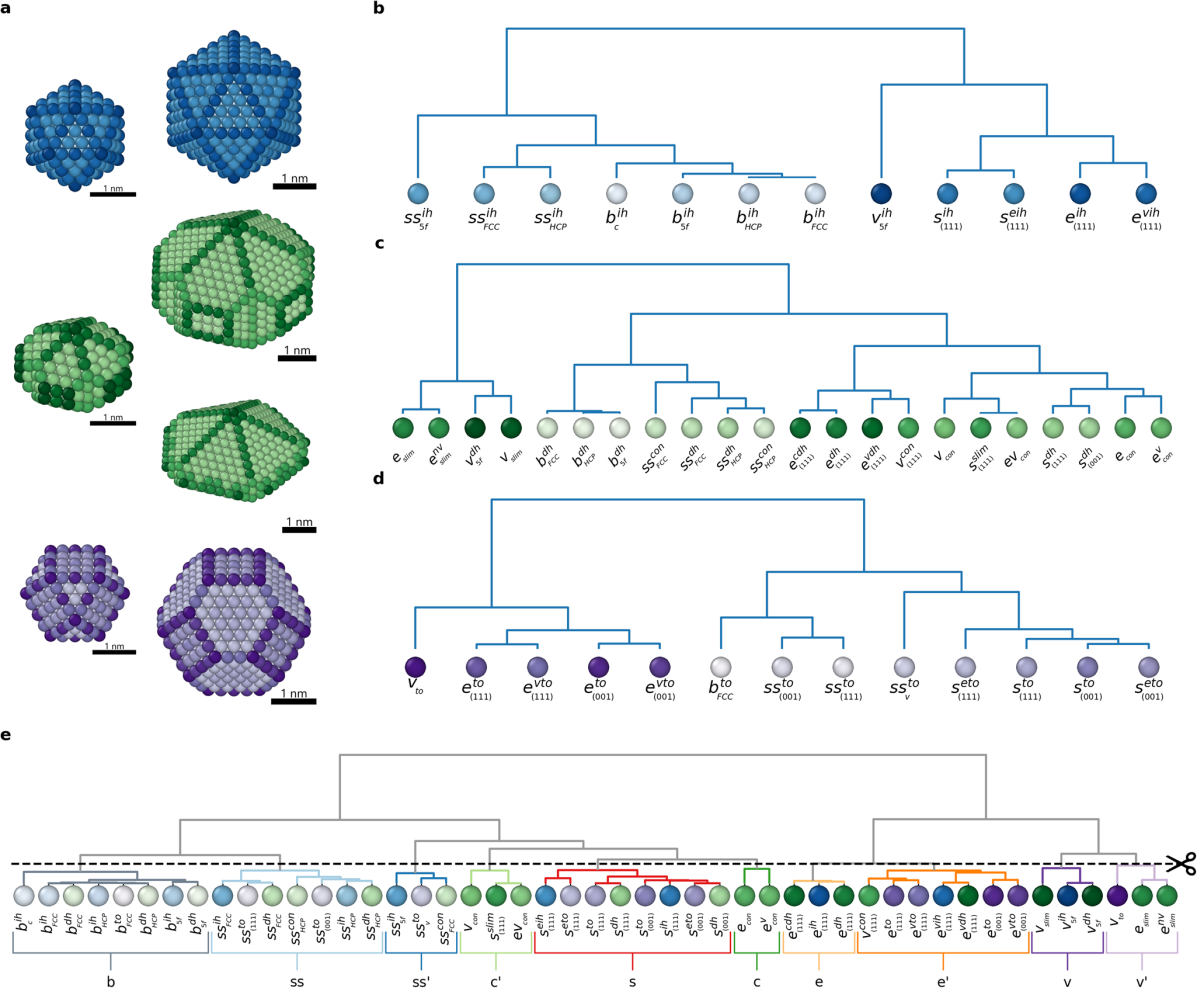
A dictionary of atomic SOAP NP environments. **a** Icosahedral (blue), decahedral (green), and truncated-octahedral Au NPs used to generate the SOAP dictionary of AEs. Together with the NPs that we simulate herein (the three on the left), also larger size NPs are included in the dictionary, in order to guarantee that this contains all AEs typical of the NP families. **b** Dendrogram connecting the various SOAP AEs proper of icosahedral NPs (blue) connected based on their SOAP distance. **c** Dendrogram for the SOAP AEs native of ideal decahedral NPs (green). **d** Dendrogram for the SOAP AEs native of ideal decahedral NPs (purple). **e** Global dendrogram connecting all the AEs of the various NP types, hierarchically classified based on their SOAP similarity using the SOAP distance. Cutting the dendrogram at a certain SOAP distance provides a coarse-grained dictionary that groups together the AEs with SOAP distance *d*_*SOAP*_ ≤ 0.08.

We created a dictionary of Au AEs (Fig. 4) that contains all AE typical of different-shape NPs. In particular, we calculated the SOAP atomic spectra of two ideal icosahedral Au NPs: *Ih*_309_, simulated in Figs. 1–3, and a larger one composed of 923 Au atoms (Fig. 4a, right: in blue). We also calculated the SOAP atomic spectra of three decahedra composed of 348 (*Dh*_348_, simulated in the next section), 1086, and 1734 Au atoms, and two truncated octahedra, composed of 309 (*To*_309_, simulated in the next sections) and 807 Au atoms (*To*_807_). In the AE dictionary, we also added an additional *To*_976_ (not included in the figure). Such a collection of NPs allowed us to maximize the number of sample AEs, obtaining a complete SOAP dictionary for *Ih*, *Dh*, and *To* Au NPs.

**Fig. 5:**
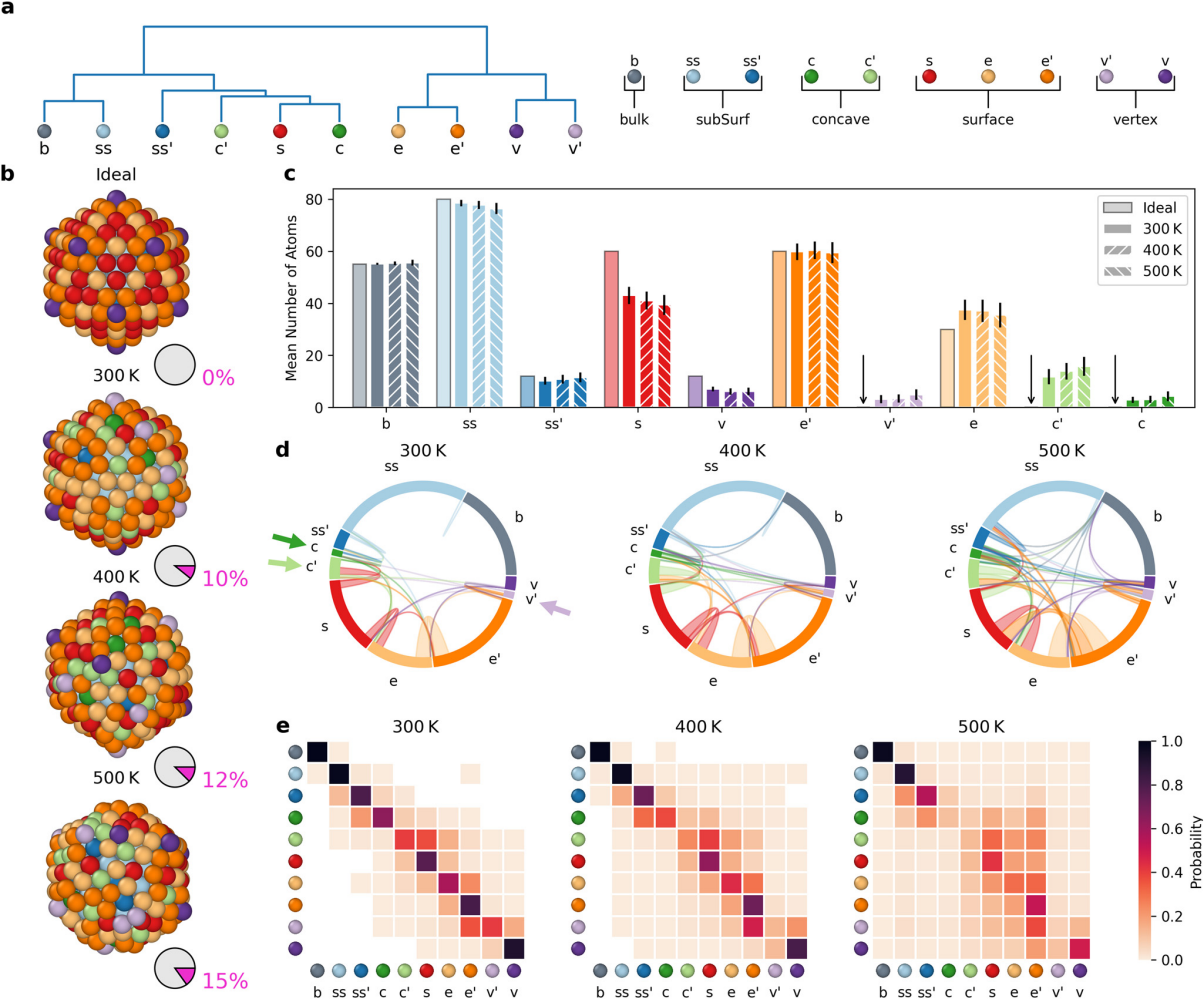
Top–down data-driven reconstruction of the innate dynamics and statistical identity of the *Ih*_309_ NP. **a** Dictionary of AEs and associated dendrogram used for the top–down analyses, obtained via cutting the complete dendrogram at *d*_*SOAP*_ = 0.08 (as shown in Fig. 4e). **b** The *Ih*_309_ NP before simulation start (top, 0 K), in steady state MD frames, taken from the MD simulations at temperature 300 K, 400 K, or 500 K (top-to-down). Coloring based on the dictionary AEs **a**: the pie charts indicate in magenta the percentage of atoms on the NP surface that do not belong to environments native to ideal icosahedral NPs. **c** Histogram counting the average number of atoms in each cluster during the last 1 μs of MD at 300 K, 400 K, 500 K (second-left to right columns for each AE), compared to the AE populations in the ideal (0 K) *Ih*_309_ NP (leftmost column for each AE). Standard deviations as vertical black lines. An arrow in place of the first column highlights the absence of certain AEs in the ideal *Ih*_309_ NP—i.e., these AEs are non-native of ideal icosahedral, and emerge with temperature (e.g., in *Ih*_309_: ${v}^{{\prime} }$, ${c}^{{\prime} }$ and *c* AEs in light-purple, light- and dark-green respectively). **d** Chord diagrams showing the interconnection between all AEs communicate with each other in *Ih*_309_ at various temperatures. Non-native AEs emerging in *Ih*_309_ are identified by colored arrows in the chord plot at 300 K (*cf*. main text for details). **e** Normalized transition matrices reporting the probabilities for atoms in the *Ih*_309_ NP at the various temperatures to remain in a given AE (*p*_*ii*_) or to exchange into another one (*p*_*i*→*j*_) in the time interval of *dt* = 1 ns.

The obtained AE dictionary contains a total of 47 different SOAP environments. Figure 4b shows the 12 ones typical of icosahedral Au NPs, organized hierarchically in a dendrogram based on their SOAP distance and similarity (see Methods). Figure 4c shows the dendrogram containing the 22 environments characteristic of the decahedral Au NP. Figure 4d shows the dendrogram of the 13 SOAP AEs typical of truncated-octahedral and cuboctahedral NPs. Finally, Fig. 4e shows the complete SOAP dictionary, containing all AEs proper of *Ih*, *Dh,* and *To* Au NPs, organized based on their SOAP distance and similarity (see Methods). Such a dictionary of SOAP spectra can be then used to compare and classify the AEs that emerge along the MD simulations of a given NP, and to understand if on that specific NP AEs emerge which are closer to those present in NPs of other shapes. While the dendrogram of Fig. 5e contains the complete information, this also shows, e.g., that most of the bulk environments across different-shape NPs are basically identical to each other. Thus, to ensure to capture relevant variations in our analysis, we opted to “truncate” the dendrogram at the distance of *d*_SOAP_ = 0.08 (and considering as relevant only differences larger than this), see Supplementary Fig. S11 and Supplementary Fig. S12 for an example on how the choice of this parameter influences the environments and the analysis of the dynamics. The cut at *d*_*SOAP*_ = 0.08 reduces the 47 AEs to 10 AEs, improving the clarity and the statistical relevance of the subsequent analysis. Nonetheless, the resolution of such analysis can be in principle adapted, based on the relevance of the difference between the AEs. This “cut” provides a coarse-grained analysis, which regroups all AEs with *d*_SOAP_ < 0.08 in macro-clusters in all SOAP environments.

In particular, the truncated dendrogram of Fig. 5a shows the final 10 AEs considered in the analysis. The *b* AE collects all NP bulk environments. The *ss* and *ss’* AEs collect all subsurface AEs: *ss* identify the AEs under the FCC(111) and FCC(001) NP faces and those under the NP edges, *ss’* identify the “non-standard" subsurface AEs under the vertexes and the convex elements. The *c* and *c’* AEs enclose the concave environments. The *s*, *e,* and *e’* enclose all surface AEs: *s* collects the AEs proper of FCC(111) and FCC(001) faces, *e,* and *e’* collects edge AEs, while *v* and *v’* those proper of vertexes. We used the “coarse-grained" SOAP dictionary for analyzing our MD simulations and distinguishing between native and non-native AEs emerging in the simulated NPs.

### A dynamic dance of native and non-native AEs shaping the surface identity of Au NPs

In Fig. 5b we show the *Ih*_309_ NP in its ideal configuration (at 0 K: after minimization) and at various temperatures (top-to-bottom: 300 K, 400 K, 500 K). In particular, the *Ih*_309_ snapshots at 300 K, 400 K, 500 K correspond to the same MD frames of Fig. 1e, and Fig. 2a, e, but in Fig. 5b the atoms are colored based on the similarity of their atomic environments and those contained in the SOAP dictionary of Fig. 5a (top–down analysis). This analysis allows us to track in detail which ones of the AEs populating the *Ih*_309_ NP at the various temperature belong to the family of the native ones, typical of icosahedral NPs, and which ones are non-native—namely, closer to those natives of different-shape NPs, such as e.g., decahedra or truncated octahedra. For each NP snapshot, a pie chart (bottom-right) shows the percentage of surface atoms belonging to native (in gray) and non-native AEs (in pink) in the *Ih*_309_. The analysis shows how the percentage of emerging non-native environments increases with increasing temperature, essentially due to increased thermal fluctuations and surface reconstructions.

The histograms of Fig. 5c report the average number of atoms belonging to each AE in the last 1 μs of MD (equilibrated-phase MD trajectories). For each AE, we represent the count with four columns (Fig. 5c): the first one refers to the AE populations in the ideal *Ih*_309_ NP, and the other three columns refer to the AE populations in the same NPs at the three simulated temperatures. The *v’* (purple), *s’* (light green), *c* (green) AEs are non-native AEs, in that these are not present in the ideal *Ih*_309_ (identified by arrows), and in icosahedral NPs in general, but emerge in *Ih*_309_ with temperature.

At each MD timestep, we know the cluster each atom belongs to so that we can track where the atoms come from and where they go in terms of AEs. This allows us to draw the chord diagrams of Fig. 5d, showing the dynamic interconnections between the various AEs populating the NP. Qualitatively, the width of the corona arcs represents the total number of transitions that happened to a given cluster during the simulation, and the chords between two clusters show the interconnections between the various AEs, the more the base of the chord is extended the more the cluster has given atoms to the one it is connected to. The color of the chords indicates the net flux (e.g., the chord connecting the red (*s*) and violet (${v}^{{\prime} }$) clusters is violet, meaning that more atoms are observed to undergo a transition in ${v}^{{\prime} }\to s$ direction than vice versa). The results qualitatively show with what AEs the non-native *v’*, *s’*, and *c* ones are primarily connected, suggesting where these non-native AEs come from and where they go. In particular, *v’* is connected with *e* edge atoms (light orange). The non-native concave *c* and *c’* AEs are connected with edge (orange), surface (red), and vertex (violet) atoms. The chord diagrams show that the exchange between the AEs increases with temperature (increasing number of chords and of chords’ widths moving left-to-right in Fig. 5d). As seen also in Fig. 1, this analysis confirms that at 300 K only surface AEs exchange dynamically, while *ss* and *b* clusters remain relatively static and separated (dynamic surface and static interior). Increasing the temperature, and in particular, at 500 K (Fig. 5d: right), the interior of the NP starts to communicate with the surface (see cyan and gray chords going towards surface AEs).

To obtain more quantitative information on the complex atomic dynamics present in the NPs, we calculated the transition probabilities for atoms belonging to such AEs to remain in or undergo a transition into the different AEs in Δ*t* = 1 ns (same analysis of Figs. 1h, Fig. 2d, h, but with this new set of top–down detected AEs). The transition matrices of Fig. 5e show that deep AEs (*b* and *ss*) have diagonal entries residence probabilities *p*_*ii*_ ~ 1 (dark colors). This confirms the rather static behavior of the interior of the NPs at all temperatures. At 500 K the blue *ss’* AE starts to communicate with the surface of the NP and, in particular, with the green *c* and *c’* concave AEs. The matrices of Fig. 5e thus show that the formation of the rosettes on *Ih*_309_ comes from such deep states, as well as (in large part) from the surface (red, *s*) and edge (*e*,*e’*) AEs. From the transition matrices of Fig. 5 it is possible to estimate, e.g., the transition probabilities, rates, timescales, and lifetimes of all these top–down detected AEs as done from that of Fig. 1h. For example, in the *Ih*_309_ NP at 300 K the non-native concave *c’* AE (light green) has a transition probability to the surface (red, *s*) AE of ${p}_{{c}^{{\prime} }\to s} \sim 0.4$, indicating a transition rate of ${k}_{{c}^{{\prime} }\to s} \sim 0.4$ ns^−1^ and characteristic transition timescale ${\tau }_{{c}^{{\prime} }\to s}=2.5$ ns (transition event observed ~ 4700 times along 1 μs of MD: see Supplementary Fig. S3). Given that in the *c’* row of the matrix the ${c}^{{\prime} }\to s$ transition is by far the fastest one, in good approximation, this allows estimating the bottom limit of the lifetime of one atom in the *c’* AE in the range of ${\tau }_{{c}^{{\prime} }} \sim 2.5$ ns. Similar estimations of the characteristic timescales for all transitions between the AEs in the NP can be easily performed from all the *p*_*i*→*j*_ reported in the transition matrices of Fig. 5e. Such analyses thus provide not only an estimation of the average composition of an NP but also, and perhaps even more interestingly for practical applications (e.g., reactivity), detailed information on the lifetime of all the native and non-native AEs populating it. In fact, the capability of an AE to activate a chemical reaction is directly related to its lifetime *vs*. by the characteristic time of the reaction itself on that AE. For example, while it is known that different atomic sites have, e.g., different reactivity and efficiency in catalyzing chemical reactions^63^, obtaining a structural/dynamical map showing how long all AEs in the NPs live (*τ*_*i*_) and how quickly they interconvert into other ones (*τ*_*i*→*j*_) is key to understand their effective efficiency in catalyzing a reaction. In fact, from a statistical point of view, if one AE has a given average lifetime *τ*_*i*_, but the characteristic timescale for the reaction to occur on that specific AE is *τ*_*react*_ > *τ*_*i*_, the probability for effectively activating the reaction on that AE would be proportional to *τ*_*i*_/*τ*_*react*_. This would provide an indication of how many times, in principle, the reactant should get in touch with the same AE to effectively activate a given reaction. Of course, performing practical estimations in this sense would require focusing on a realistic case and also estimating the reactivity of all visited AEs in the NP. While this is not the main point of this paper, this is certainly feasible, which underlines the potential of the approach. Moreover, we stress that such a purely probabilistic interpretation stands as far as the reactive species do not significantly alter the dynamics and features of the AEs present on the NP—e.g., no or negligible chemisorption (if such a condition does not hold, a proper reactive parametrization and simulation of the system is needed, where new AEs may appear on the NP surface upon interaction with the reactants)^64^.

As to what is seen in the matrix of Fig. 2h, the matrix of Fig. 5e (right) shows that at 500 K the surface of *Ih*_309_ NP is basically pre-melted^61^ (*p*_*ii*_ of surface AEs < 0.5, meaning that in Δ*t* = 1 ns the atoms in those AEs have a higher probability to move to another AE, rather than to remain in the same one). This indicates that the entire *Ih*_309_ has atomic dynamics faster than the nanosecond scale (liquid-like dynamics). On the other hand, at the resolution of our analyses, the atomic dynamics on the NP surface appear more “discrete” at 300 K and 400 K (solid-like dynamics).

**Fig. 6:**
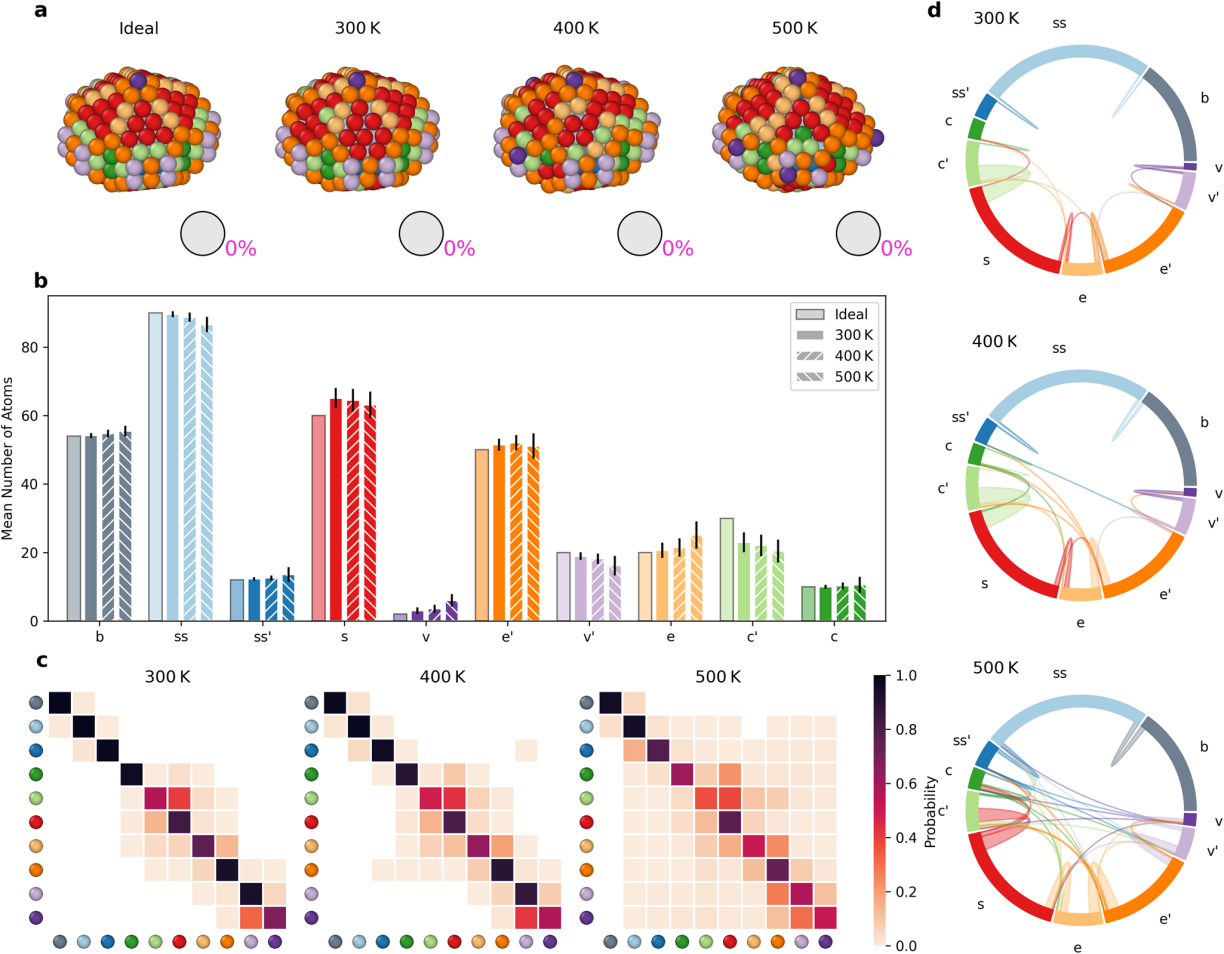
Top–down analysis of AEs in the *Dh*_348_ NP. **a** Snapshots of the ideal *Dh*_348_ (0 K) and at 300 K, 400 K, and 500 K. Atoms within the NPs are colored based on the AEs dictionary. **b** Histogram counting the average number of atoms in each AE during the last 1 μs of MD simulation at 300 K, 400 K, 500 K (second-left to right columns for each AE). Standard deviations as vertical black lines. **c** Normalized transition matrices reporting the probabilities for atoms in the *Dh*_348_ NP to remain in a given AE (*p*_*ii*_) or to exchange into another one (*p*_*i*→*j*_) in the time interval of *dt* = 1 ns at the various temperatures. **d** The chord diagrams show the dynamic interconnections between all AEs detected in the NP at different temperatures.

We repeated the same analysis for the *Dh*_348_ NP (Fig. 6). The data show that this NP is more stable than *Ih*_309_ at all simulated temperatures. This NP has been chosen because its ideal conformation shows at least one atom per each cluster in the set that we identified with the cut. Consequently, this results in the pie charts of Fig. 6a showing always 0% pink. At 300 K and 400 K, the internal *b* and *ss* AEs are not in communication with the surface. At 500 K, some communication arises but also at such high temperatures in this case the atomic dynamics on the NP surface appear as “discrete" and closer to that of *Ih*_309_ at 300 K and 400 K (solid-like dynamics).

**Fig. 7:**
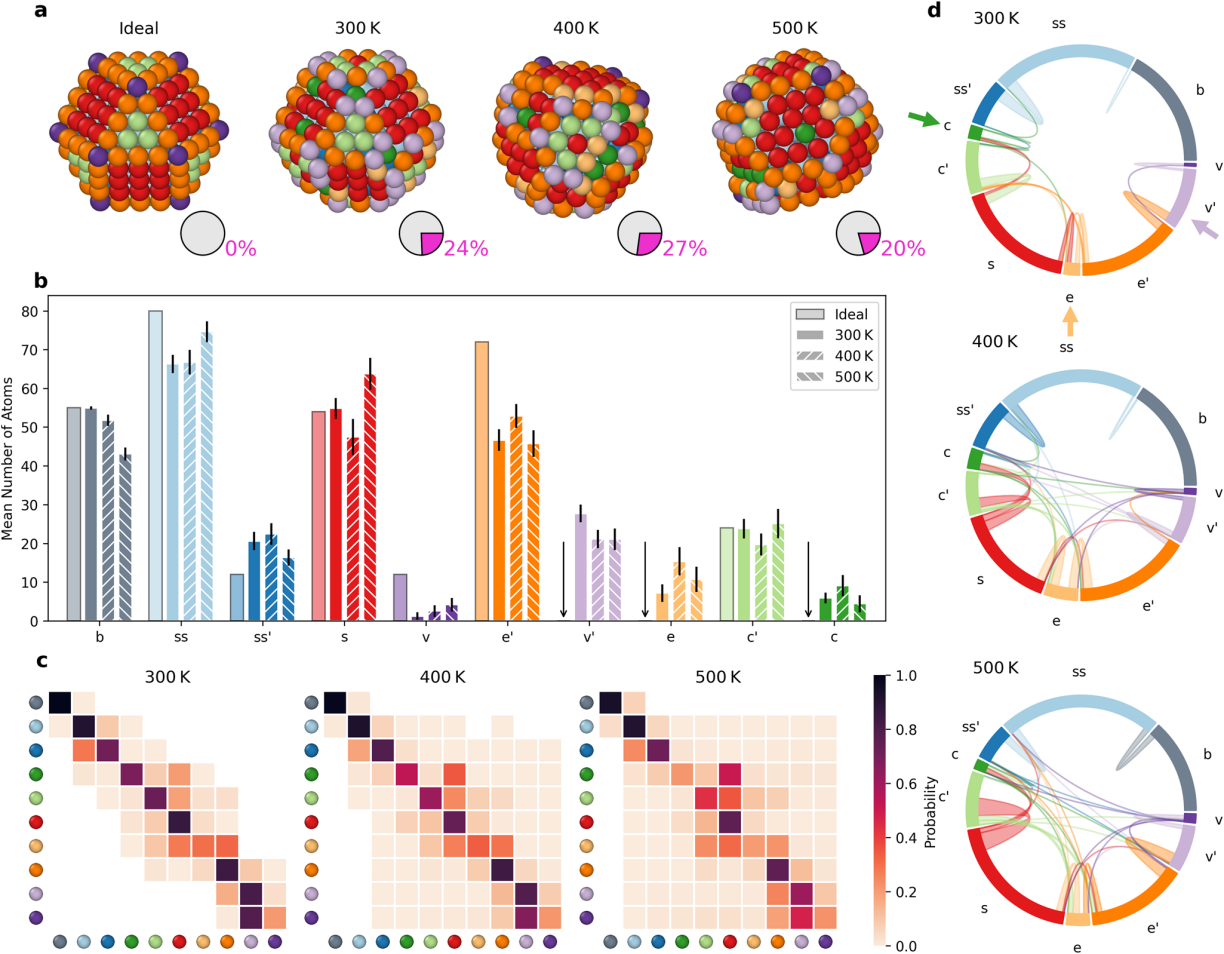
Top–down analysis of AEs in the *To*_309_ NP. **a** Snapshots of the ideal *To*_309_ (0 K) and at 300 K, 400 K, and 500 K. Atoms within the NPs are colored based on the AEs dictionary (the pie charts indicate in magenta the percentage of atoms on the NP surface that does not belong to environments native to ideal truncated-octahedral or cuboctahedral NPs. **b** Histogram counting the average number of atoms in each AE during the last 1 μs of MD simulation at 300 K, 400 K, 500 K (second-left to right columns for each AE). Standard deviations as vertical black lines. **c** Normalized transition matrices reporting the probabilities for atoms in the *To*_309_ NP to remain in a given AE (*p*_*ii*_) or to exchange into another one (*p*_*i*→*j*_) in the time interval of *dt* = 1 ns at the various temperatures. **d** The chord diagrams show the dynamic interconnections between all AEs detected in the NP at different temperatures.

Among the investigated NPs, *To*_309_ (Fig. 7) is one of the most interesting. In fact, the *To*_309_ arrangement is known to be a non-favorable FCC arrangement. Indeed, *To*_309_ is more dynamic than *Dh*_348_ at all investigated temperatures (see also Supplementary Movie 3). Interestingly, at 300 K this NP is found less stable and more dynamic than *Ih*_309_, with ~24% of its surface atoms in non-native AEs (pie charts in Fig. 6a and histograms of Fig. 6b). However, at 500 K, *To*_309_ is found more stable than *Ih*_309_ and its surface shows “discrete" atomic dynamics. Raw transition matrices for *Dh*_348_ and *To*_309_ are provided in Supplementary Fig. S4 and S5.

We compared the chord diagrams obtained with the top–down and bottom-up analysis (see Supplementary Fig. S10) to evaluate their consistency in terms of fluxes. The results show that the two analyses are consistent in this regard. However, comparing actual kinetics is not straightforward because the AEs identified by the two analyses are not directly related. The top–down analysis defines AEs based on a dictionary, while the bottom-up analysis derives them from the MD trajectories. Moreover, the consistency between the two analyses strongly depends on the cutoff for the dendrogram in the top–down analysis. Both analyses are therefore complementary and provide different insights into the atomic dynamics of Au NP’s.

Altogether, these results show that such analysis is transferable and flexible. In particular, this can be used *(i)* to obtain a thorough characterization of the complex atomic dynamics of the NPs that is difficult to attain with other approaches, and *ii* to compare and classify different types of metal NPs based on the AEs that emerge and populate their structure and on their complex dynamics.

## Discussion

Understanding the intricate atomic dynamics on the surface of metal NPs in relevant regimes is paramount for unveiling the physical chemistry and diverse properties of the nanoparticles. This characterization, while fundamental, typically poses significant challenges, both experimentally and computationally. In this work, we achieve such a detailed characterization *via* the use of a concerted ML approach that includes a combination of a bottom-up and top–down data-driven analyses of steady-state MD trajectories of various types of Au NPs. In the first step, the bottom-up ML analysis detects in a purely data-driven way the main atomic environments (AEs) that populate an NP in MD steady state based on the levels of order/disorder and the structural similarity/dissimilarity between them as captured by high-dimensional SOAP data extracted from the MD trajectories, and further dimensionality reduction and unsupervised clustering (Figs. 1 and 2). The choice of the SOAP descriptor, having a defined distance metric, allows us to perform advanced analysis techniques, i.e., density based clustering, to better understand and classify the atomic environments at the surface. Tracking the individual atoms along the MD and classifying them based on the detected SOAP environments allows for resolving the complex atomic dynamic that is present on the NP surface at different temperatures (see transition matrices in Figs. 1, 2). In particular, this allowed us to identify in a data-driven unbiased way all the AEs that populate the NPs in the MD local equilibrium and to understand where these come from and where they go in terms of interconversion into other AEs. On a second step, a top–down data-driven classification based on the similarity/distance between/from the SOAP spectra of each atom at each sampled step of the steady state MD trajectories and the SOAP spectra characteristic of a variety of different Au NPs contained in a SOAP spectra dictionary. Such a SOAP dictionary contains the SOAP spectra of all AEs proper of ideal, e.g., icosahedral, decahedral, and truncated-octahedral Au NPs, which allows us to learn which ones of the AEs appearing on the surface of a given simulated NP in MD steady state are “native” of that type of NP, and which ones are “non-native” (i.e., typical of ideal NPs of a different shape). We repeat such analysis on three types of different-shape Au NPs (Figs. 5–7: i.e., *Ih*_309_, *Dh*_348_, and *To*_309_). Notably, such analysis allows us not only to estimate the “statistical equivalent identity” of the various NPs—namely, what do the NPs look-like in relevant dynamic regimes (histograms of Figs. 5–7) –, but also obtain relevant dynamic information as on the average lifetime and interconnection rates of all visited atomic (native and non-native) environments present on their surface (transition matrices in Figs. 5–7). While the dictionary of AEs presented herein is based on the analyzed set of Au NPs, it can be easily expanded to include more particle geometries. Nevertheless, the provided analysis already shows the generality of such an approach, i.e., it can be applied to any kind of metal NP. Moreover, our analysis can be easily applied without further tuning to metallic systems in which vacancies arises, i.e., on that case the user would just need to add some new environments to the AE dictionary.

With our methodology, we achieve an unparalleled atomistic-level understanding of the behavior of Au NPs at various temperatures, a level of detail rarely achieved in experimental studies due to the challenges associated with tracking individual atomic motions over time. The in-depth understanding of the dynamic properties of atomic sites populating the surface of Au NPs, as presented in this study, is of significant practical relevance. Particularly, it can inform the design of more effective NP-based heterogeneous catalysts, enhancing the efficiency of industrial processes. We envisage that this may offer a useful platform for predictions—e.g., for estimating whether the NP’s reactivity in given conditions will be likely to increase or decrease if the conditions change (e.g., changing the temperature), based on the changes in the intrinsic dynamics of the atoms within the NP and the survival timescales of all the AEs populating it. While the examples provided for catalytic applications are speculative, they underscore the broader implications of our analysis. The detailed understanding of the intrinsic structural dynamics of NPs under application conditions, as provided here, has indeed far-reaching potential. For instance, this knowledge could be instrumental in optimizing the performance of Au NPs not only in catalysis but also in sensor devices and biomedical applications, where their unique physical and chemical properties are highly valued.

We expect that the approaches that we present herein will see a broad application in many other cases, opening new routes towards the discovery of structural-dynamic-property relationships of a variety of similar metal NP systems.

## Methods

### Atomistic models and MD simulations of the NPs

The atomistic models for the *Ih*_309_, *Dh*_348_, and *To*_309_ NPs were built with the tool “clusterCreator"^65^. Preliminary basin hopping calculations showed that, at these sizes, Au favors the formation of decahedral NPs, followed by the icosahedron and the cuboctahedron (see Supplementary Table S2). To simulate the NPs, we used the SMATB^56–58^ potential available in LAMMPS^66^ (Supplementary Table S1)^43^. The NP models were initially minimized using the built-in command in LAMMPS (set up with etol = 10^−6^ ftol = 10^−8^, maxiter = 1000, and maxeval = 10000), then we performed a small thermalization of 20000 MD steps with the timestep set to 1 fs on the NP with the velocities initialized to the desired temperature and with the thermostat with the same settings of the main simulation. We then simulated different Au NPs at temperatures of 300 K, 400 K, and 500 K. All MD simulations were conducted in the canonical ensemble using the LAMMPS’s Langevin thermostat, using a timestep of 5 fs, and a damping parameter for the Langevin thermostat set to 100 ps. We simulated each NP system for a total of 2 μs of MD. During the simulations, all NP systems reached a steady state in the MD regime (equilibrium). All our analyses were thus conducted on 1000 frames taken every 1 ns along the last 1 μs of each MD simulation, during which the populations of all detected AEs plateaued (see Supplementary Fig. S6–S9).

### SOAP analysis

We used the SOAP^50^ as high-dimensional abstract descriptors of the local atomic environment that surround each atom in the NPs during the simulations. The SOAP spectra of each atom in the NPs (Fig. 1a) were calculated at each of the 1000 MD snapshots taken from the last 1 μs of the simulations (every 1 ns), using the atomic positions as they are, without any preprocessing procedure. We thus come out with SOAP datasets containing a total of 309,000, 348,000, or 309,000 SOAP spectra for *Ih*_309_, *Dh*_348_, and *To*_309_ simulated systems respectively, at each temperature. We used *dscribe*^67^ to generate the SOAP vectors with the following parameters: *r*_cut_ = ~ 4.48 Å (corresponding to 110% of the Au FCC lattice parameter, which includes in the calculation up to the first two neighbors in FCC, and up to the third in the HCP case even in case of some small local fluctuations). We set up the *l*_max_ parameters for the spherical harmonics to 8, and the *n*_max_ parameter to set up the number of radial basis functions to use to 8. With these parameters, the SOAP spectrum for each atom is a vector of 576 components (of which 324 are unique).

### Bottom-up analysis: PCA and clustering

We then reduced the dimensionality of our SOAP vectors by projecting the normalized SOAP vector on the principal components (PCs) using the PCA algorithm from scikit–learn^68^, and taking into consideration only the first three PCs, hence reducing the number of components from 576 to 3. This allows retaining ~ 99% of the total variance in the SOAP datasets. We computed the PCA using the data from the *Ih*_309_ at 300 K simulation, and then we projected the data from the simulations at the other temperatures on the computed PCs. In particular, we applied the clustering method HDBSCAN*^54^ (we set up HDBSCAN* with the following parameters: min_cluster_size=125, cluster_selection_method="eom") to the first 3 PCs of the data from *Ih*_309_ 300 K simulation to identify the main AEs visited by the atoms during the simulation. We then used the trained algorithm to predict the clusters in the other simulations, by applying the trained predictor on the first 3 PCs of the simulations *Ih*_309_ at 400 K, and *Ih*_309_ at 500 K. Note that the analysis presented in Fig. 1 is not the direct result of the learning on the training set (the *Ih*_309_ 300 K), but the application of the trained prediction algorithm to that training set, to have a more homogeneous procedure with the other two datasets of *Ih*_309_ at the higher temperatures presented in Fig. 2. The prediction procedure classifies some of the data points as noise, for the sake of this paper we extracted the ’exemplar’ data points from the learning set, each one assigned to its cluster, and then we assigned each of the noise points to the cluster of the ’euclidean closest’ exemplar point. In Fig. 1b, c we show the environment (learned on the simulation of *Ih*_309_ at 300 K) that the SOAP+PCA+HDBSCAN* procedure predicted on the ideal *Ih*_309_ structure.

### Top–down analysis: similarity, distance, and dictionary

For the analysis, we presented in Figs. 4–7, we used the well–defined SOAP distance^51^ to classify the environments visited during the simulations, as done in other previous works^47–49^. The SOAP distance between two SOAP spectra $\overrightarrow{a}$ and $\overrightarrow{b}$ is calculated as:

1 $${d}_{SOAP}\left(\overrightarrow{a},\overrightarrow{b}\right)=\sqrt{2-2{{{{{{{\mathscr{K}}}}}}}}\left(\overrightarrow{a},\overrightarrow{b}\right)}$$

where, with the SOAP power spectrum representation that we are using,

$${{{{{{{\mathscr{K}}}}}}}}\left(\overrightarrow{a},\overrightarrow{b}\right)=\frac{\overrightarrow{a}\cdot \overrightarrow{b}}{\left\Vert \overrightarrow{a}\right\Vert \left\Vert \overrightarrow{b}\right\Vert }.$$

To apply such a classification we built a dictionary: we attempted to create the most complete dictionary for icosahedral, decahedral, and octahedral NPs’ AEs by choosing the most different environments from various minimized Au NPs. To enrich our dictionary, together with the NPs that we effectively simulated in this work, we also included larger-size NPs possessing a higher variety of AEs in their ideal state. We obtained a dictionary of 47 elements. To simplify its usage, we hierarchically classified its elements using the hierarchical clustering algorithms implemented in scipy^69^. First of all, we used Equation (1) for calculating the distance between each of the environments belonging to the dictionary. Then we created a binary tree that represents this classification by using the “*complete*” algorithm for hierarchical clustering, which at each step couples the closest elements in the set and assigns to the newly formed couple the largest distance from each remaining element of the set, and uses the new distance in the next steps until it has completed the classification. We represent this tree in the dendrogram in Fig. 4e, where we show that we have chosen to apply a cut at the distance of 0.08 [*dSOAP*]. This cut leads to the creation of 10 different groups of dictionary entries (that can be seen more clearly in Fig. 5a) with similar geometrical characteristics, from the original 47 environments. During the MD simulations analysis, we assigned an environment to one of these 10 clusters in two steps. The first step is to classify it as one of the 47 elements of the original environment dictionary: we do this simply by assigning it to the closest element of the dictionary in terms of the SOAP distance (using Equation (1)). The second step is to classify our analyzed environment by assigning it to the cluster to which its closest reference belongs.

### Temporal analysis

We calculated the transition matrices from the cluster information of each atom along the simulation^45,47^. Transition matrices are calculated by accumulating a table whose elements are the number of transitions *i*→*j* or *i*→*i* in the main diagonals that happen at each time step. We then obtain the probability for an atom to undergo a transition into another specific AE (or of remaining in the same AE) after each timestep (in *dt* = 1 ns in our analyses) by normalizing each row to 1. In the figures in which we show a transition matrix, we decided to represent with blank squares the transitions not observed.

## Supplementary information

Supplementary Information

Description of Additional Supplementary Files

Supplementary Movie 1

Supplementary Movie 2

Supplementary Movie 3

## Data Availability

Details on the analysis procedures and for the simulations’ setup, are provided in the Methods section and in the Supplementary Information file. Additional images on the non-normalized transition matrices, and on the attained steady-states during the MD are provided in the Supplementary Information file. Complete data and materials pertaining to the atomistic simulations and data analyses conducted herein (input files, model files, raw data, analysis tools, etc.) are available at: 10.5281/zenodo.8108900. Other information needed is available from the corresponding author upon reasonable request.
