# Supplementary material for: Machine learning of atomic dynamics and statistical surface identities in gold nanoparticles
Source: Commun Chem. 2023 Jul 5;6:143. doi: 10.1038/s42004-023-00936-z (PMC10322832; doi:10.1038/s42004-023-00936-z)
Supplement: Supplementary file 1 — Supplementary Information [file 42004_2023_936_MOESM1_ESM.pdf]

Supporting information for: Machine Learning of Atomic Dynamics and Statistical Surface Identities in Gold Nanoparticles

Daniele Rapetti[1], Massimo Delle Piane[1], Matteo Cioni[1], Daniela Polino[2], Riccardo Ferrando[3], and Giovanni M. Pavan[1,2,*]

[1]Department of Applied Science and Technology, Politecnico di Torino, Corso Duca degli Abruzzi 24, 10129 Torino, Italy
[*]corresponding author: Giovanni M. Pavan (giovanni.pavan@polito.it)
[3]Department of Physics, Università degli Studi di Genova, Via Dodecaneso 33, 16146 Genova, Italy
[2]Department of Innovative Technologies, University of Applied Sciences and Arts of Southern Switzerland, Polo Universitario Lugano, Campus Est, Via la Santa 1, 6962 Lugano-Viganello, Switzerland

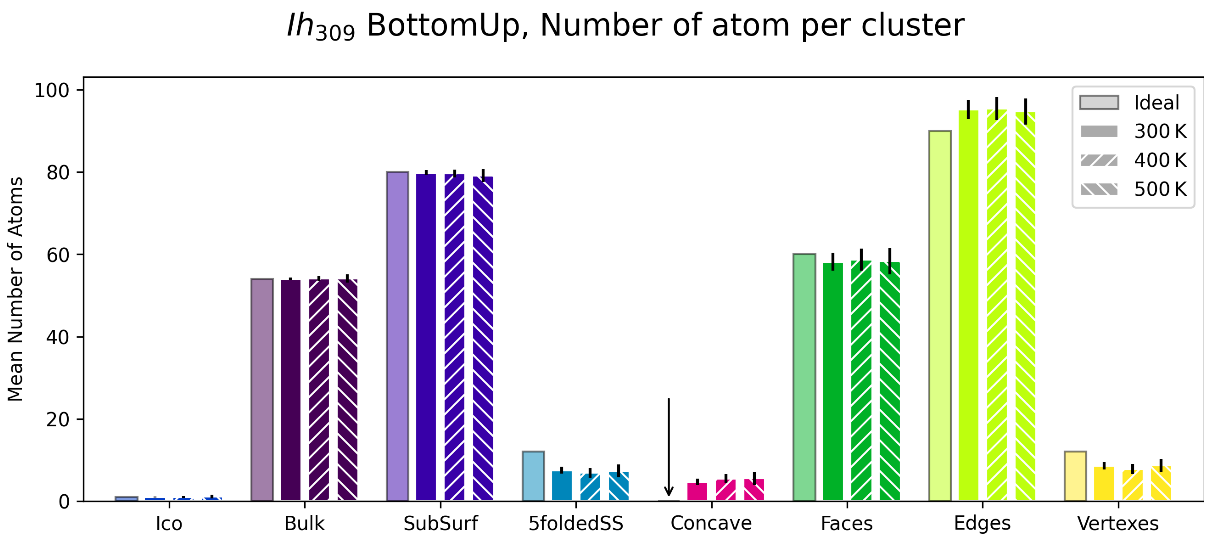

Figure S1. Histograms showing the average number of atoms per cluster (AE), calculated for the simulations Ih_{309} at 300 K, 400 K, and 500 K analyzed with the *bottom-up* classification.

radius	p	q	A	Ξ	c_{start}	c_{end}
2.88	10.35	4.178	0.210	1.818	4.07293506	4.9883063257983666

Table S1. Au parameters used in the simulations (SMATB potential).

cluster	Energy	ΔE from Lowest
Ih_{309}	-1125.87 eV	+3.16 eV
Ih_{309} With3Rosette	-1126.86 eV	+2.17 eV
To_{309}	-1125.3 eV	+3.73 eV
Best309($\sim Dh$)	-1129.03 eV	0

Table S2. Energies of the ideal 309 Au clusters, for an Au_{309} near the global minimum configuration, and an Ih_{309} with 3 rosettes making an anti-Mackay face.

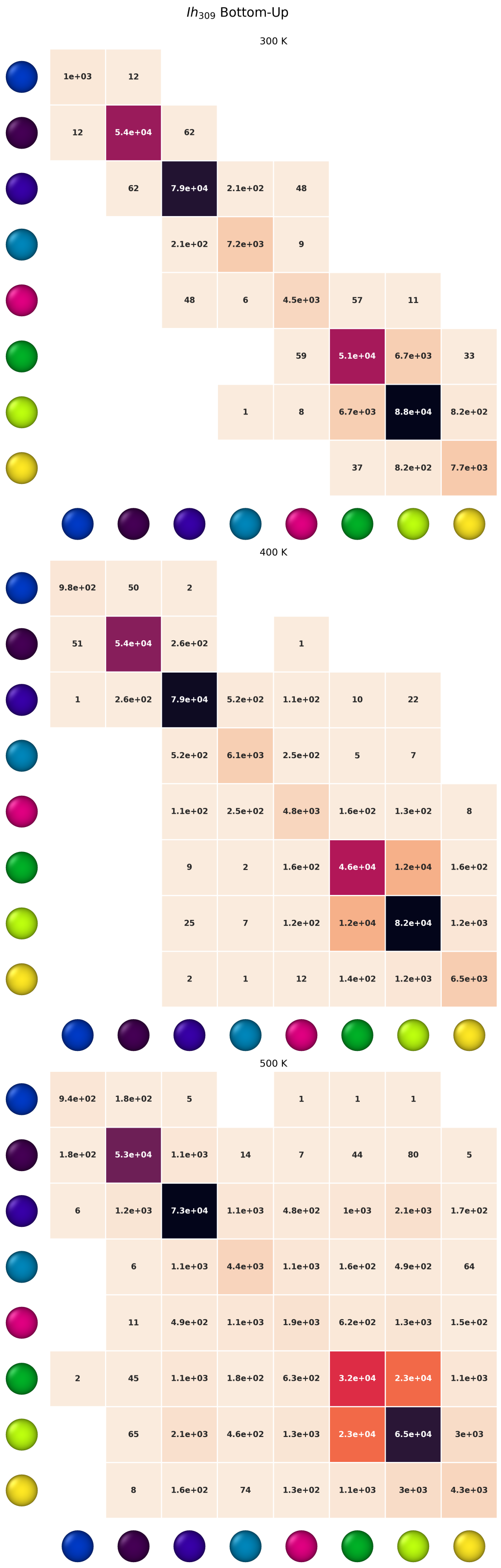

Figure S2. Raw transitions matrices reporting the number times a transition event is registered along the MD simulations of the Ih_{309} NP at 300 K, 400 K, and 500 K analyzed with the *bottom-up* classification (same matrices normalized by rows are reported in Figures 1 and 2).

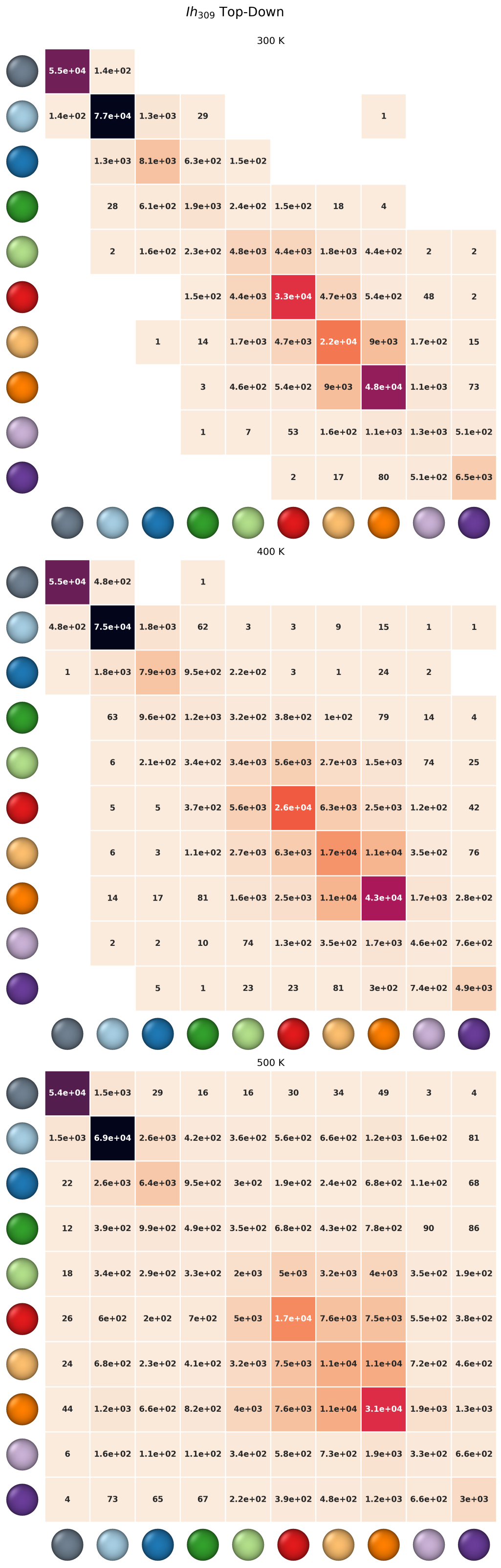

Figure S3. Raw transitions matrices reporting the number times a transition event is registered along the MD simulations of the Ih_{309} NP at 300 K, 400 K, and 500 K analyzed with the *top-down* classification (same matrices normalized by rows are reported in Figure 5).

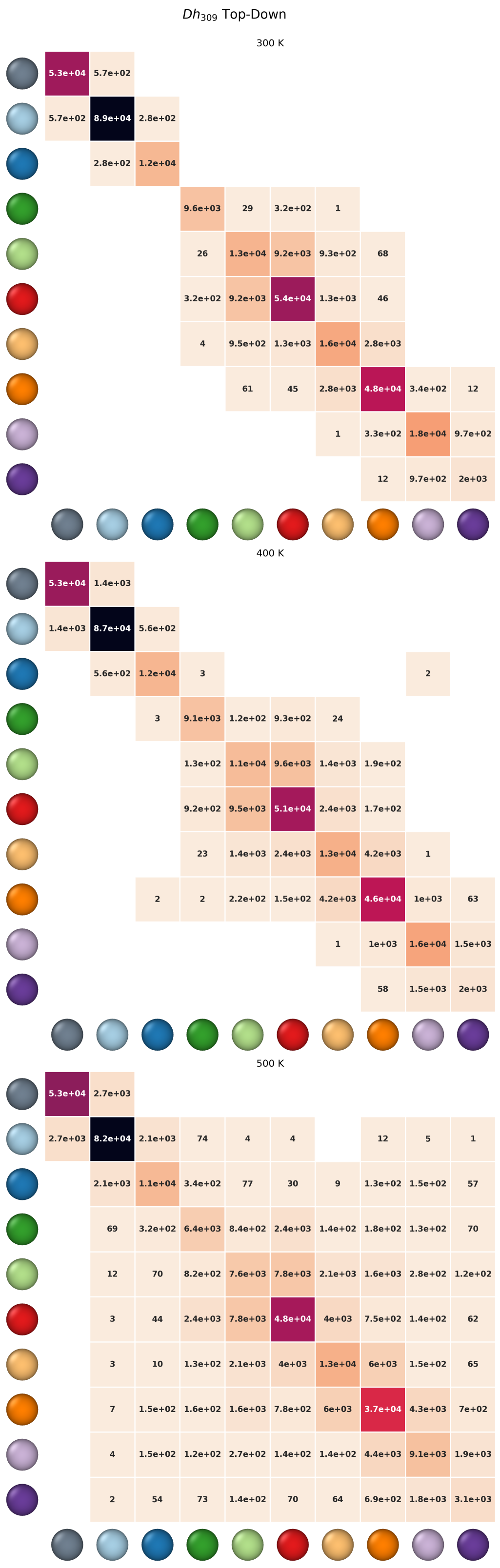

Figure S4. Raw transitions matrices reporting the number times a transition event is registered along the MD simulations of the Dh_{348} NP at 300 K, 400 K, and 500 K analyzed with the *top-down* classification (same matrices normalized by rows are reported in Figure 6).

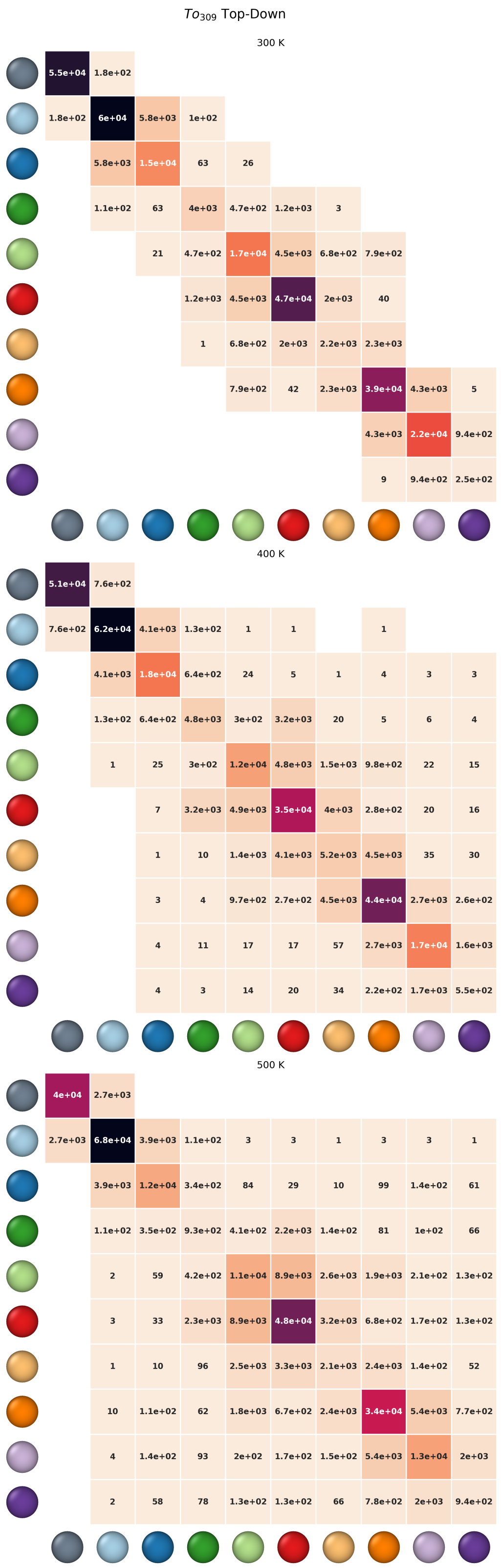

Figure S5. Raw transitions matrices reporting the number times a transition event is registered along the MD simulations of the To_{309} NP at 300 K, 400 K, and 500 K analyzed with the *top-down* classification (same matrices normalized by rows are reported in Figure 7).

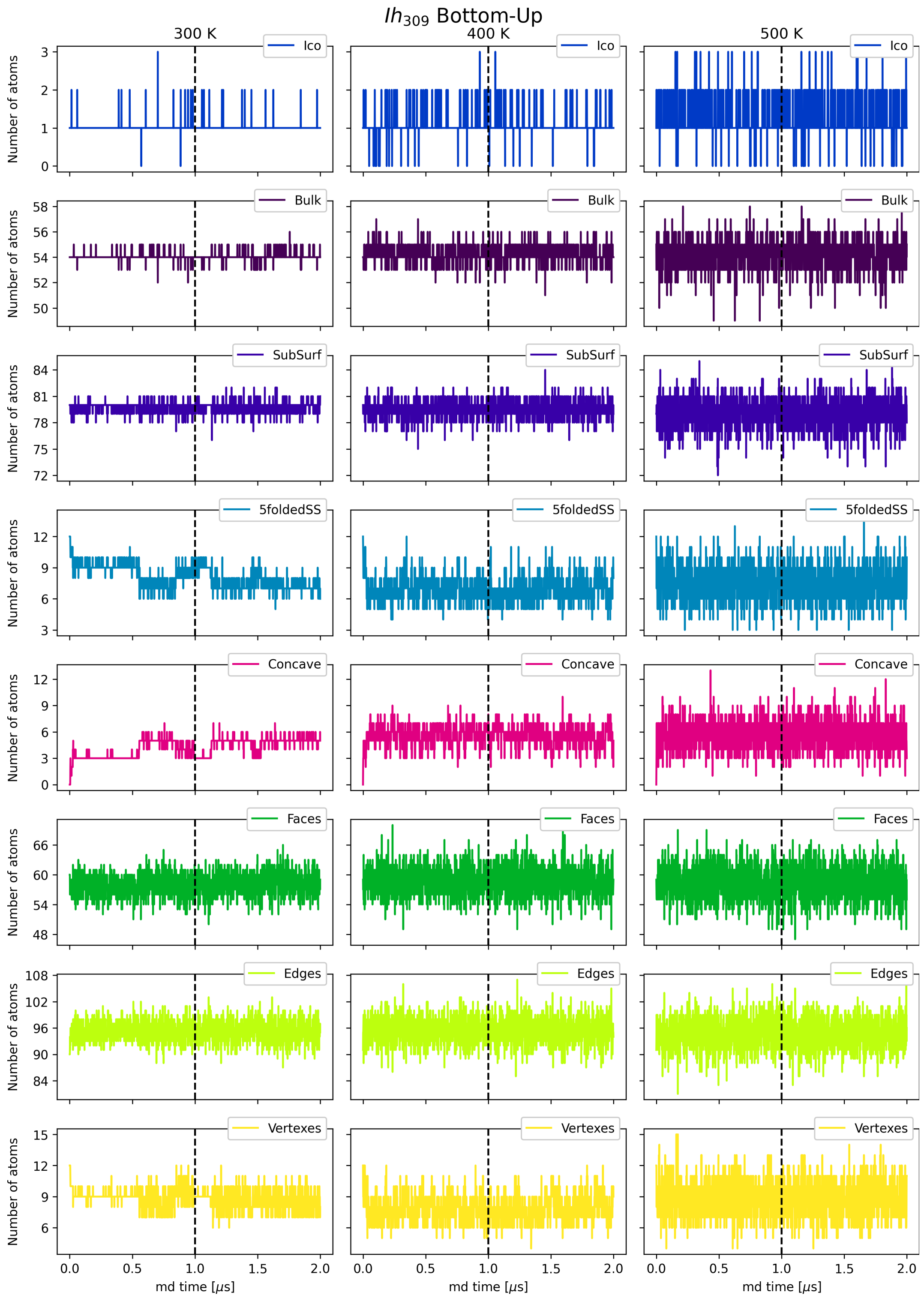

Figure S6. Histograms (number of atoms) for the AEs detected *bottom-up* in the MD simulations (2 μs) of the Ih_{309} NP at the various simulated temperatures. The vertical dotted lines represent the start of the data analysis window (*i.e.*, the last 1 μs of MD).

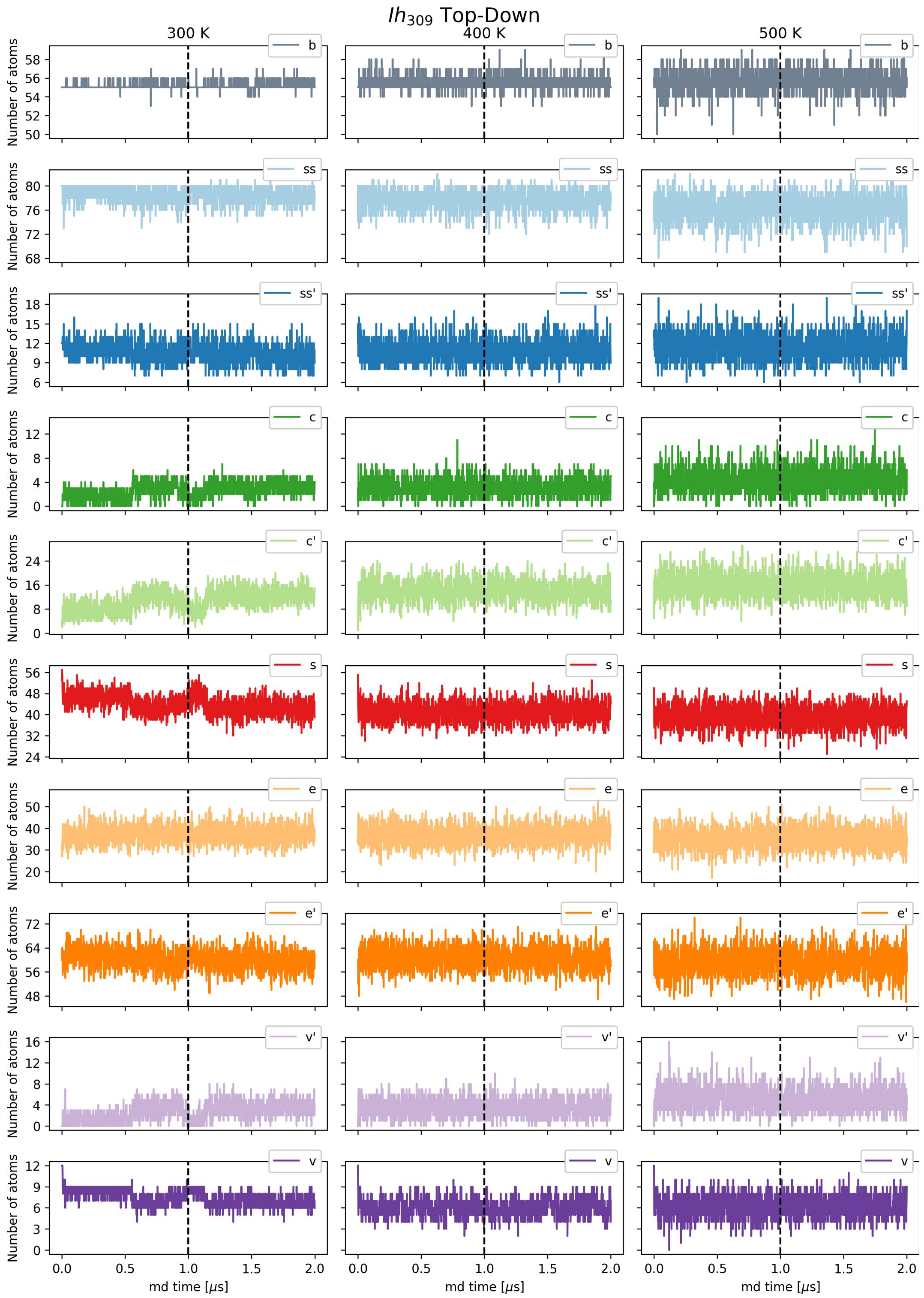

Figure S7. Histograms (number of atoms) for the AEs detected *top-down* in the MD simulations (2 μs) of the Ih_{309} NP at the various simulated temperatures. The vertical dotted lines represent the start of the data analysis window (*i.e.*, the last 1 μs of MD).

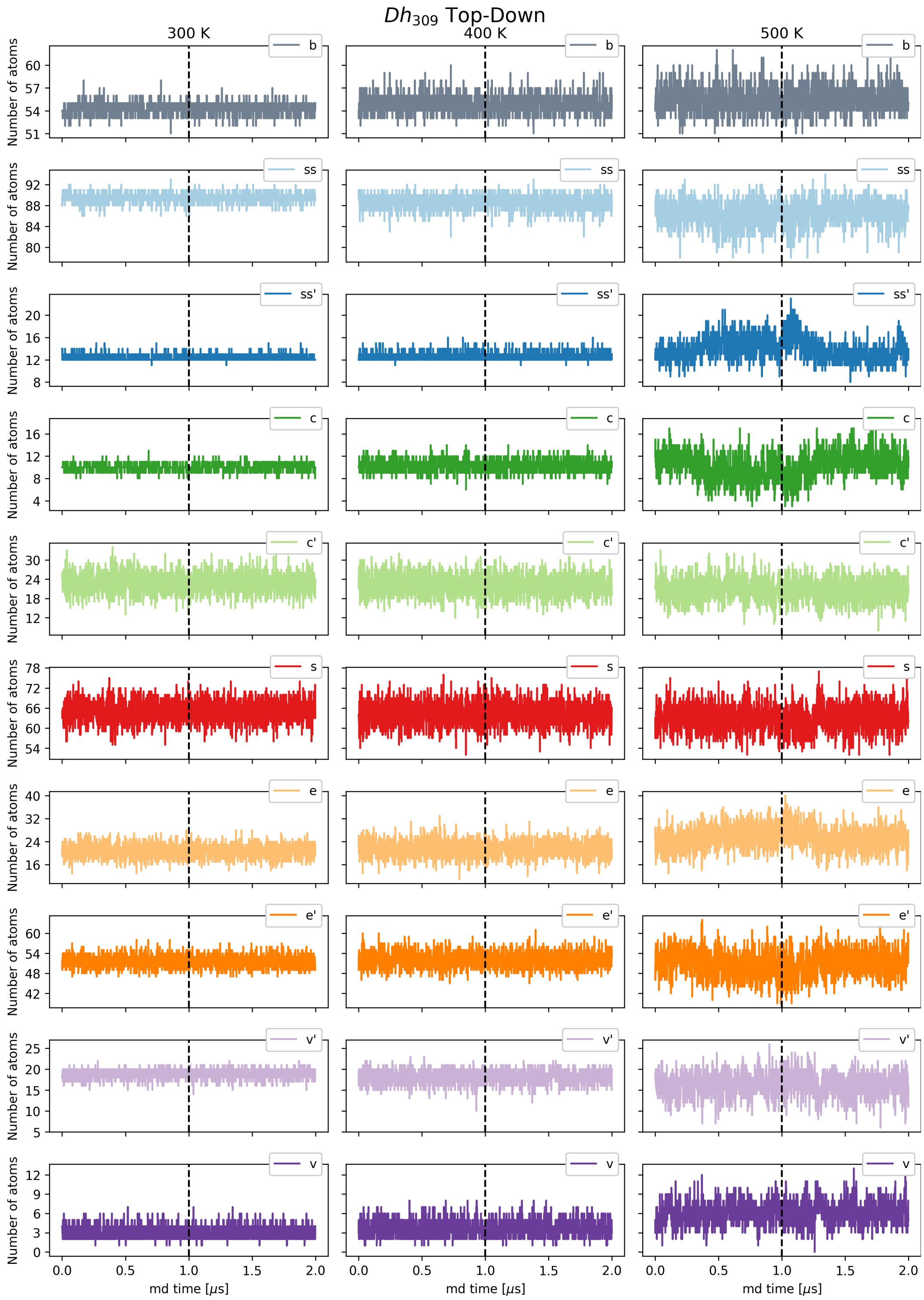

Figure S8. Histograms (number of atoms) for the AEs detected *top-down* in the MD simulations (2 μs) of the Dh_{348} NP at the various simulated temperatures. The vertical dotted lines represent the start of the data analysis window (*i.e.*, the last 1 μs of MD).

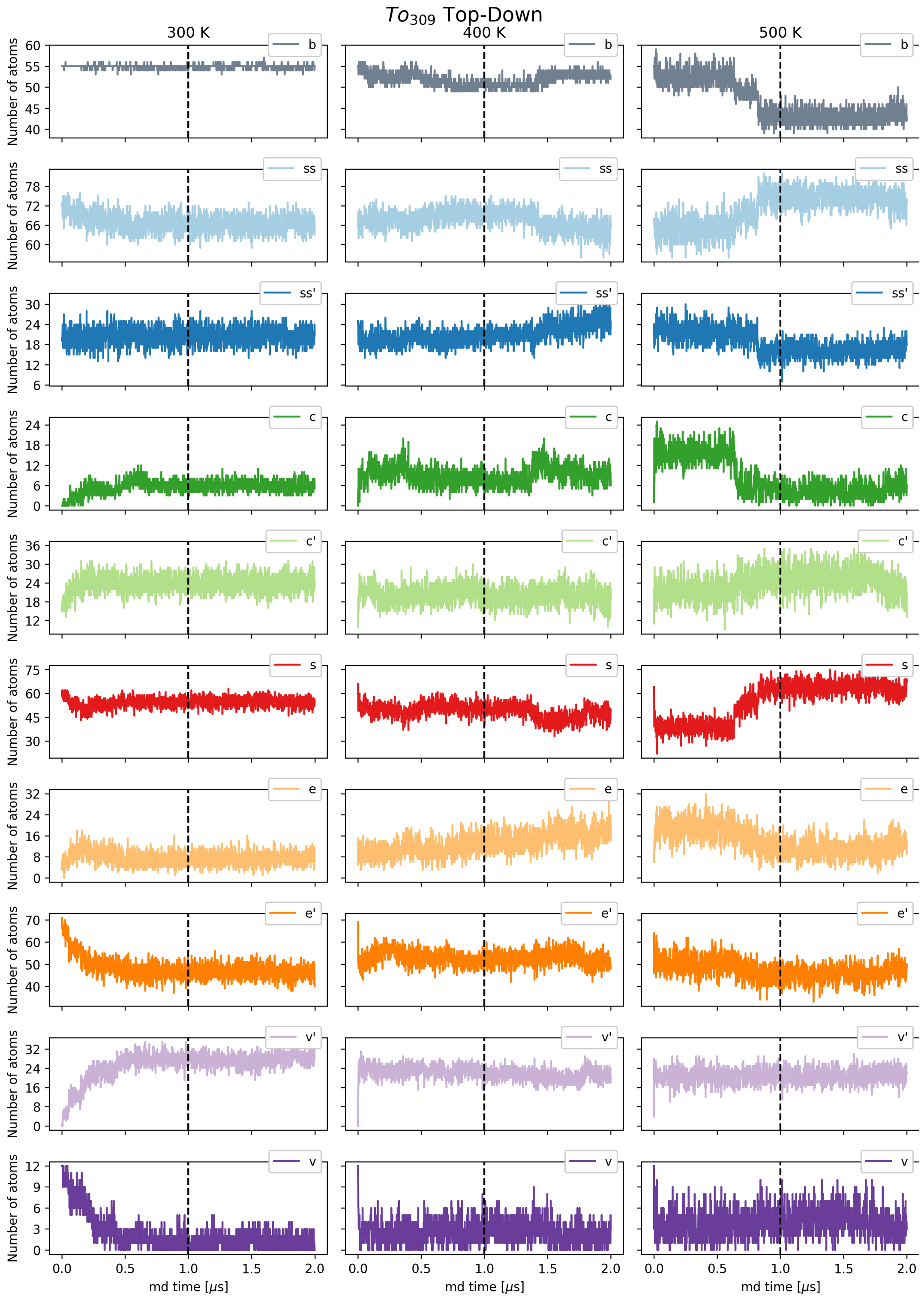

Figure S9. Histograms (number of atoms) for the AEs detected *top-down* in the MD simulations (2 μs) of the To_{309} NP at the various simulated temperatures. The vertical dotted lines represent the start of the data analysis window (*i.e.*, the last 1 μs of MD).

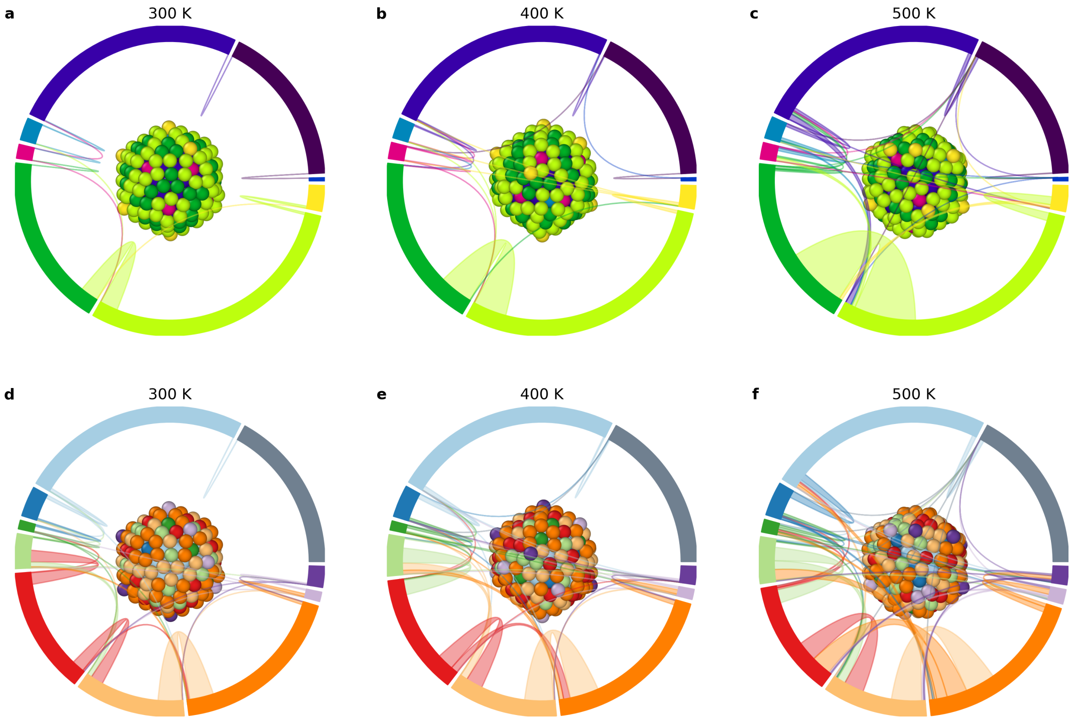

Figure S10. A parallel of the net fluxes in the Ih_{309} at various temperatures, for the BU and the TD analyses. The two analyses are not completely overlapping due to a missing one-to-one relationship between the AEs found in the BU analysis and the AE selected in the TD analysis.

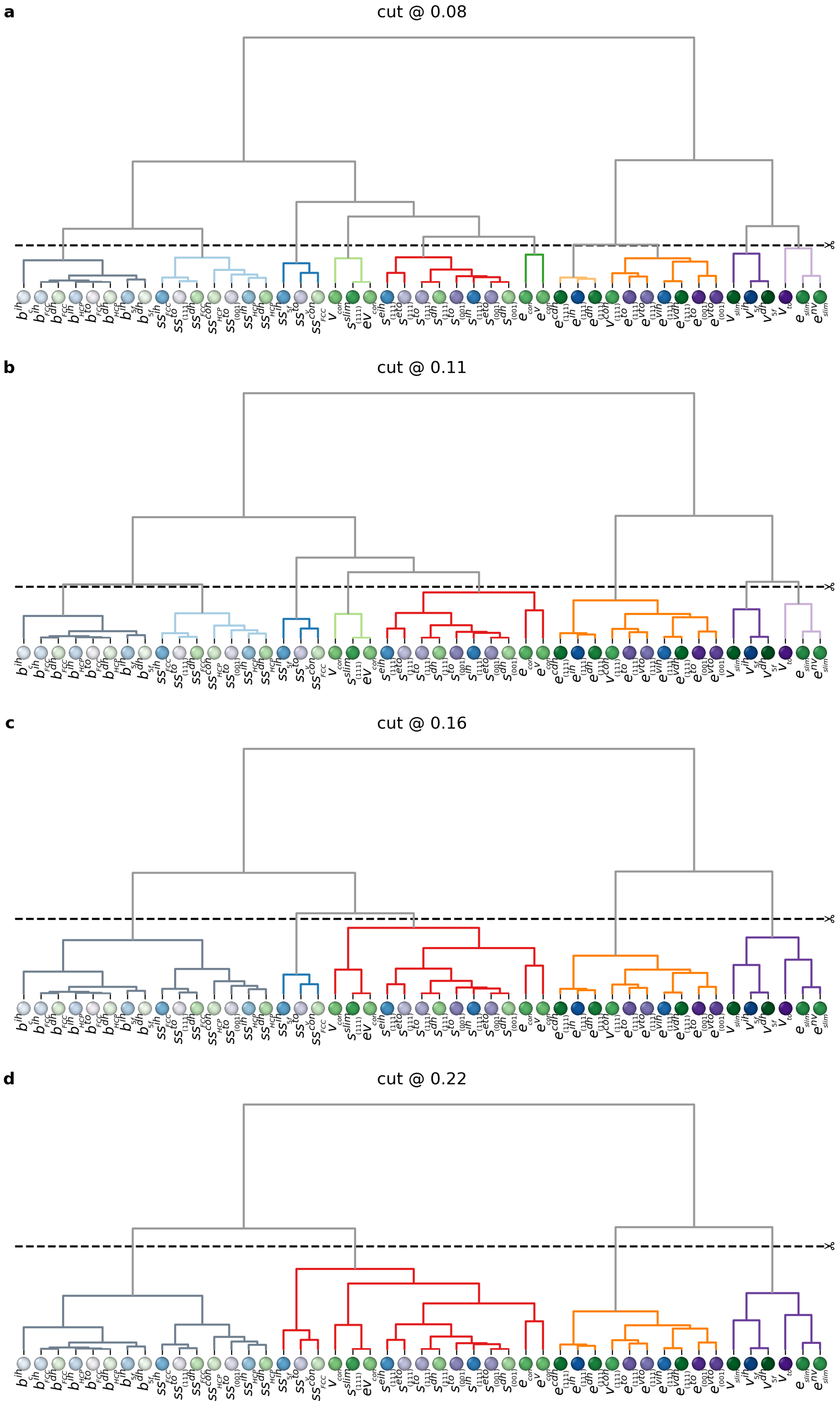

Figure S11. The influence of the chosen cut (0.22, 0.17, 0.11 and the used 0.08) on the dendrogram of Figure 4: by increasing the cut distance we reduce the number of clusters, and so we lose details about the geometry of the AEs.

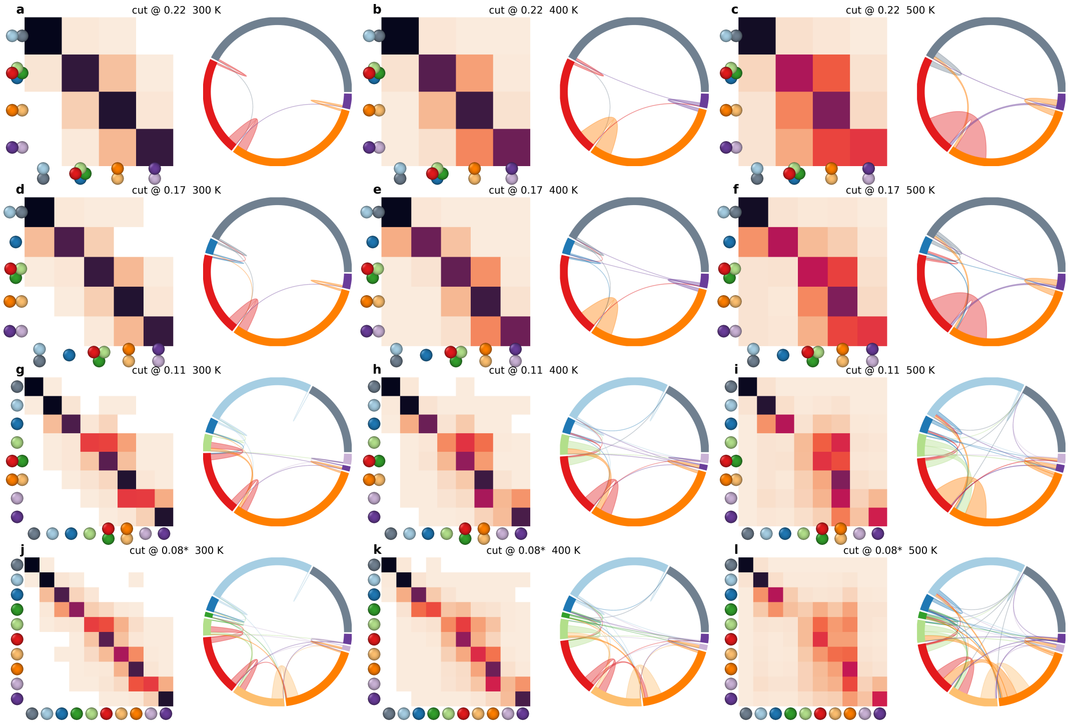

Figure S12. The influence of the number of AEs due to the chosen cut (0.22, 0.17, 0.11 and the used 0.08) on the transition probability and on the net flux for the Ih_{309} at various temperatures. In the transition matrices we show the grouped AEs from the cut=0.08 that we used in the main analysis
