# Supplementary material for: Machine learning of atomic dynamics and statistical surface identities in gold nanoparticles
Source: Commun Chem. 2023 Jul 5;6:143. doi: 10.1038/s42004-023-00936-z (PMC10322832; doi:10.1038/s42004-023-00936-z)
Supplement: Supplementary file 2 — Description of Additional Supplementary Files [file 42004_2023_936_MOESM2_ESM.pdf]

Description of Additional Supplementary Files

File name: Supplementary Movie 1

Description: MD of the Ih309 NP at 300K, colored according to both the bottom up and top down analyses.

File name: Supplementary Movie 2

Description: MD of the Ih309 NP at 400K, colored according to the bottom up analysis.

File name: Supplementary Movie 3

Description: MD of the Dh348 NP at 400K, colored according to the top down analysis.
